# Using ChatGPT-4 to Create Structured Medical Notes From Audio Recordings of Physician-Patient Encounters: Comparative Study

**DOI:** 10.2196/54419

**Published:** 2024-04-22

**Authors:** Annessa Kernberg, Jeffrey A Gold, Vishnu Mohan

**Affiliations:** 1 Department of Medical Informatics and Clinical Epidemiology Oregon Health and Sciences University Portland, OR United States

**Keywords:** generative AI, generative artificial intelligence, ChatGPT, simulation, large language model, clinical documentation, quality, accuracy, reproducibility, publicly available, medical note, medical notes, generation, medical documentation, documentation, documentations, AI, artificial intelligence, transcript, transcripts, ChatGPT-4

## Abstract

**Background:**

Medical documentation plays a crucial role in clinical practice, facilitating accurate patient management and communication among health care professionals. However, inaccuracies in medical notes can lead to miscommunication and diagnostic errors. Additionally, the demands of documentation contribute to physician burnout. Although intermediaries like medical scribes and speech recognition software have been used to ease this burden, they have limitations in terms of accuracy and addressing provider-specific metrics. The integration of ambient artificial intelligence (AI)–powered solutions offers a promising way to improve documentation while fitting seamlessly into existing workflows.

**Objective:**

This study aims to assess the accuracy and quality of Subjective, Objective, Assessment, and Plan (SOAP) notes generated by ChatGPT-4, an AI model, using established transcripts of History and Physical Examination as the gold standard. We seek to identify potential errors and evaluate the model’s performance across different categories.

**Methods:**

We conducted simulated patient-provider encounters representing various ambulatory specialties and transcribed the audio files. Key reportable elements were identified, and ChatGPT-4 was used to generate SOAP notes based on these transcripts. Three versions of each note were created and compared to the gold standard via chart review; errors generated from the comparison were categorized as omissions, incorrect information, or additions. We compared the accuracy of data elements across versions, transcript length, and data categories. Additionally, we assessed note quality using the Physician Documentation Quality Instrument (PDQI) scoring system.

**Results:**

Although ChatGPT-4 consistently generated SOAP-style notes, there were, on average, 23.6 errors per clinical case, with errors of omission (86%) being the most common, followed by addition errors (10.5%) and inclusion of incorrect facts (3.2%). There was significant variance between replicates of the same case, with only 52.9% of data elements reported correctly across all 3 replicates. The accuracy of data elements varied across cases, with the highest accuracy observed in the “Objective” section. Consequently, the measure of note quality, assessed by PDQI, demonstrated intra- and intercase variance. Finally, the accuracy of ChatGPT-4 was inversely correlated to both the transcript length (*P*=.05) and the number of scorable data elements (*P*=.05).

**Conclusions:**

Our study reveals substantial variability in errors, accuracy, and note quality generated by ChatGPT-4. Errors were not limited to specific sections, and the inconsistency in error types across replicates complicated predictability. Transcript length and data complexity were inversely correlated with note accuracy, raising concerns about the model’s effectiveness in handling complex medical cases. The quality and reliability of clinical notes produced by ChatGPT-4 do not meet the standards required for clinical use. Although AI holds promise in health care, caution should be exercised before widespread adoption. Further research is needed to address accuracy, variability, and potential errors. ChatGPT-4, while valuable in various applications, should not be considered a safe alternative to human-generated clinical documentation at this time.

## Introduction

Medical documentation is an integral aspect of clinical practice, ensuring accuracy and comprehensive patient management and serving as a communication tool among health care professionals. In recent years, it has become increasingly evident that inaccuracies in medical notes lead to miscommunication, diagnostic discrepancies, and patients’ perceptions of subpar medical care [1]. Beyond the immediate implications of documentation errors, documentation demands have been identified as a significant contributor to physician burnout [2]. With health care professionals spending an increasing amount of their working hours on paperwork, there is less time and energy left for direct patient care.

To counter this, many institutions have adopted the use of intermediaries, such as medical scribes or speech recognition software, to shoulder the documentation load and allow clinicians to focus on patient interactions. However, both of these solutions have significant limitations and concerns regarding documentation accuracy and lack of impact on many provider-specific metrics surrounding after-hours charting and burnout [3,4]. In addition, the financial implications of employing medical scribes render them inaccessible to numerous health care practices [5]. Consequently, there is a continued search for innovative solutions to create effective and accurate documentation while seamlessly integrating into existing workflows.

With the rapid and exponential growth in computing capacity, artificial intelligence (AI) is being increasingly used in health care, holding the promise of revolutionizing medical documentation, thus potentially alleviating the burden on physicians [6]. AI-powered systems can analyze vast amounts of data quickly, identify patterns, and suggest diagnostic options. Although the allure of AI is undeniable, questions regarding its accuracy, reliability, and suitability in the clinical setting remain. The maturation of speech recognition technology has led to large-scale adoption by health care organizations, allowing for real-time transcription services. This, combined with software using large language models (LLMs), now enables the creation of structured medical notes in close temporal relation to the clinical encounter, thereby decreasing the clinician documentation burden [7,8]. Multiple software vendors are developing and deploying documentation assistance software powered by ambient AI, referred to as ambient digital scribes. There is already significant interest on the part of clinicians and health care organizations to adopt them. However, little data exist on the safety and quality of the documentation, with analysis made more difficult by the proprietary AI engine used by each vendor.

One such AI system is OpenAI’s ChatGPT-4, a state-of-the-art LLM known for its ability to engage in text-based communication with users (as a chatbot), which is used in some commercial ambient digital scribe solutions. Released in November 2022, ChatGPT-4 is trained on a vast amount of text data from the internet and uses an LLM to answer the users’ prompts. Health care providers envision numerous applications for ChatGPT-4, such as answering patient questions, automated insurance prior authorizations, and creating differential diagnoses [9,10]. It is important to note that open AI platforms, such as ChatGPT-4, are not recommended for clinical use due to the many regulatory and privacy issues. Despite this, there is a continued interest in whether ChatGPT-4 could serve as a freely available tool to assist as a documentation intermediary, bridging the gap between health care professionals and the tedious task of recordkeeping.

However, it is imperative that prior to the widespread adoption of these tools, their safety and efficacy need to be evaluated in a structured and clinically contextually relevant manner. Therefore, the goal of this study was to use transcripts from simulated patient-provider encounters to determine the accuracy, readability, and reproducibility of ChatGPT-4–generated Subjective, Objective, Assessment, and Plan (SOAP) notes.

## Methods

### Overview

As part of a project designed to evaluate the accuracy and efficacy of human scribe–generated notes, we created 14 simulated patient-provider encounters. All encounters used professional standardized patients and represented a wide range of ambulatory specialties. A standardized patient is an individual trained to simulate a medical scenario for health care education, assessment, and research. Briefly, for each case, a storyboard was created by subject matter experts and used for training the standardized patient to ensure standard content delivery according to best practices [11]. After an initial dry-run, each scenario was conducted in a simulated ambulatory patient exam room equipped with audio-video capture. At the end of the scenario, audio-video files were exported for use. These cases represented a variety of diagnoses (Table 1).

Audio files for each case were professionally transcribed. For each encounter, a list of key reportable elements was created for each case using the transcripts and informed by the initial storyboard, by 2 clinical experts of the study team. This is being used as the scoring rubric for subsequent analysis. These encounter transcripts were then fed into ChatGPT-4 using a standard prompt (“generate a clinical note in SOAP format for the following”). The SOAP format is a widely used clinical documentation format that concatenates data elements of the clinical interview into headers representing SOAP-related components. The SOAP format is a standard model for medical documentation, providing a clear, concise framework for health care professionals to record and share patient information. Each transcript was run through the model three times to assess output fidelity associated with replicability, thus generating three documentation versions for each case for a total of 42 ChatGPT-4 generated SOAP notes (the prompt and full output are present in Multimedia Appendix 1). A new discussion space was created for each case to prevent the various transcripts from conflating each other. Each prompt request was conducted consecutively within the same discussion space.

After acquiring the generated notes, various comparisons were made to assess the output’s accuracy and quality. Within a single case, the 3 versions were analyzed based on errors generated. A list of errors was defined as follows: (1) omissions—where expected documentation elements or data were missing; (2) incorrect—where the data element was referred to but incorrect; and (3) additions—information added that was not in the transcript. The framework for defining quality in clinical documentation based on omissions, incorrect information, and additions is a structured approach to evaluate the accuracy and completeness of medical records. These characteristics (omissions, incorrect information, and additions), if present, help define the quality of documentation given their implications. For example, omissions can lead to gaps in patient care, misdiagnosis, or delays in treatment. Incorrect information can compromise patient safety and lead to negative health consequences. Additions, while not always harmful, can be inaccurate and reduce the efficiency of care delivery. This framework is particularly useful in assessing the performance of health care documentation processes, such as those generated by medical scribes, and in quantifying appropriate information retrieval [4,12]. A correct data element was defined as one without the previously outlined errors.

**Table 1:** Case number and associated diagnosis (14 simulated patient-provider encounter transcripts representing a variety of diagnoses).

Case number	Diagnosis
1	Gastroenteritis
2	Incarcerated inguinal hernia
3	Diabetic ketoacidosis
4	Ovarian cyst
5	Pneumonia
6	Menstrual migraine
7	Breast mass
8	Heart failure
9	Polymyalgia rheumatica
10	Congestive heart failure
11	Decreased fetal movement
12	Diverticulitis
13	Scleroderma
14	Colon cancer

To ensure and assess note quality, we outlined critical data elements for each clinical case. Members of the study team independently selected these crucial data elements and subsequently compiled them to guarantee comprehensiveness. They used these elements to generate a gold standard checklist and an associated gold standard History and Physical Examination note. Then, 2 raters graded the 3 ChatGPT-4 versions of each encounter based on whether they correctly included, missed, or wrongly presented the corresponding data element. We enumerated the number of errors and correct data elements for each version. Afterward, we compared the correct data elements across the 3 ChatGPT-4 versions for presence and consistency, as follows: (1) across all three versions, (2) across two versions, (3) only in a single version, or (4) not present at all. Finally, we compared the percentages of appropriate data elements across the versions to the transcript’s length and the number of data elements.

The data elements were divided into three documentation-related sections: (1) *Subjective*, further subdivided into the history of present illness and other patient-reported information, including medications, allergies, family history, social history, and past medical history; (2) *Objective*, which includes vital signs, physical exam, and any reported test results; and (3) *Assessment* and *Plan*, which includes the provider reported differential, plan, and follow-up instructions. The percentages of correct data elements were then compared based on these categories.

Lastly, the Physician Documentation Quality Instrument (PDQI) scoring system, which is a validated tool to assess note quality, was used to evaluate the quality of the generated notes [13]. Using a set of predefined criteria, the PDQI facilitates the objective analysis of documentation practices. Within the PDQI, 9 criteria assess if the document is (1) up to date, (2) accurate, (3) thorough, (4) useful, (5) organized, (6) comprehensible, (7) succinct, (8) synthesized, and (9) consistent. The items are then scored based on a 5-point Likert scale, with the highest value representing the ideal characteristic. A maximum score of 45 represents the document that *extremely* shows the associated attribute, and a minimum score of 9 points indicates that the attribute is *not at all* present. The PDQI score was calculated for the 3 versions of the generated note, averaged, and compared across the 14 cases by a member of the study team (AK).

### Statistical Analysis

All statistical analyses were performed using GraphPad Prism (version 10; GraphPad Software Inc). For between-group comparisons, we used the Friedman test for overall and between-group comparisons given the nonnormal distribution of the data as determined by the Kolmogorov-Smirnov test. Pearson *r* test was used for univariate correlations. A *P* value <.05 was considered statistically significant.

### Ethical Considerations

The study was deemed exempt from an institutional review board approval as it did not include human subjects and therefore did not pose any risks.

## Results

**Figure 1:**
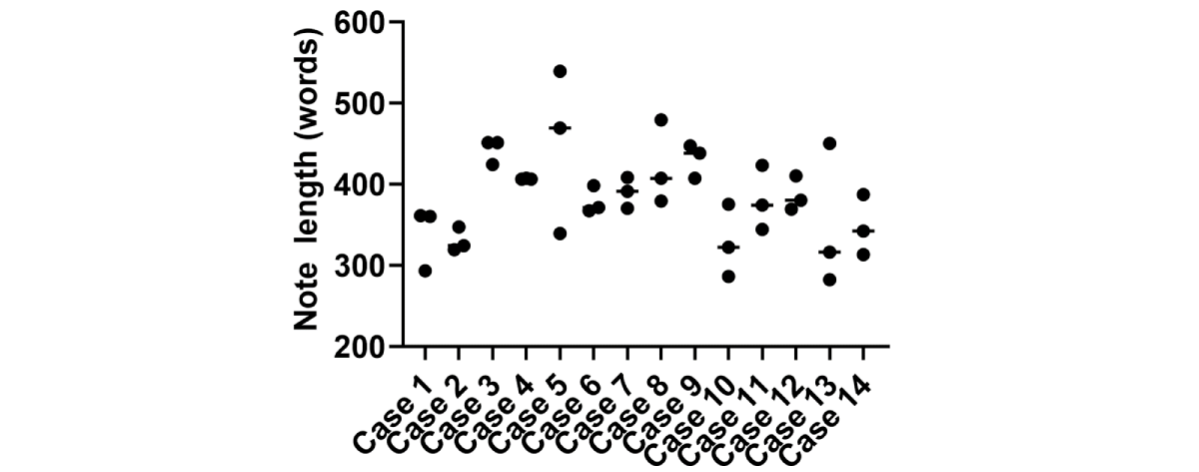
ChatGPT-4–generated note length per case (a comparison of the 14 cases versus the ChatGPT-4–generated note lengths).

We first looked at the overall structure of the notes. Consistently, ChatGPT-4 was able to generate a SOAP-style note. Overall, there was a significant variance in note length between the 3 replicates, with the transcripts of some cases being very similar in length, while others showed nearly a 50% variance between replicates (Figure 1).

**Figure 2:**
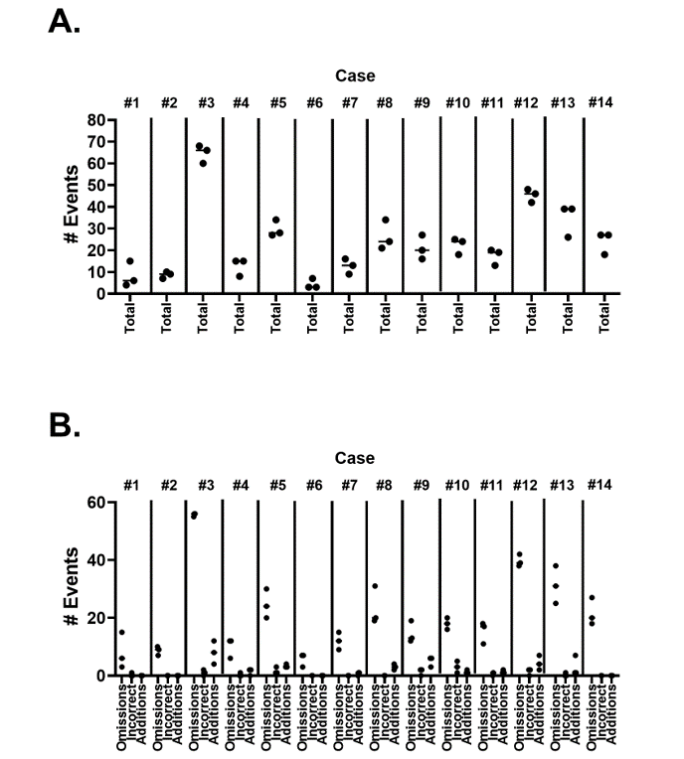
Accuracy of ChatGPT-4–generated notes (variations in errors). (A) The 3 ChatGPT-4–generated note replicates were compared based on the total number of error events per case and based on (B) omissions, incorrect facts, and addition errors per case.

We classified errors into 3 types: errors of omission, those related to incorrect facts, and errors associated with information addition. Overall, the total number of errors ranged from 5.7 to 64.7 errors per case, with significant differences between the replicates (Figure 2A). When we subdivided errors into the 3 basic types, errors of omission were by far the most common, comprising, on average, 86.3% of all errors, followed by addition errors (10.5%) and incorrect facts (3.2%). Examples of these types of errors are illustrated in Table 2. There was significant variance both in the total number and distribution of errors between cases and between replicates of the same case (Figure 2B).

**Table 2:** Error examples (examples of omission, incorrect facts, and addition errors seen in the generated notes).

Type of error and case			Example of error
**Omissions**			
	Case 2: incarcerated inguinal hernia	Failed to mention the lack of appetite or blood in vomit on review of systems	
	Case 5: pneumonia	Failed to recommend admission to the hospital.	
	Case 10: congestive heart failure	Failed to report echo results.	
**Incorrect facts**			
	Case 5: pneumonia	Reported a regular heart rate when it was tachycardic. Stated the decision to admit to the hospital would be made later when admission was recommended now.	
	Case 11: decreased fetal movement	Added the fetus was measuring 3 weeks behind expected gestational age when the fetus was measuring smaller than expected with no quantification	
**Additions**			
	Case 7: breast mass	Stated weight loss was intentional when this was not mentioned.	
	Case 8: heart failure	Added the patient was noncompliant with medication when compliance was not mentioned.	
	Case 9: polymyalgia rheumatica	Added additional labs and consults that were not mentioned.	

**Figure 3:**
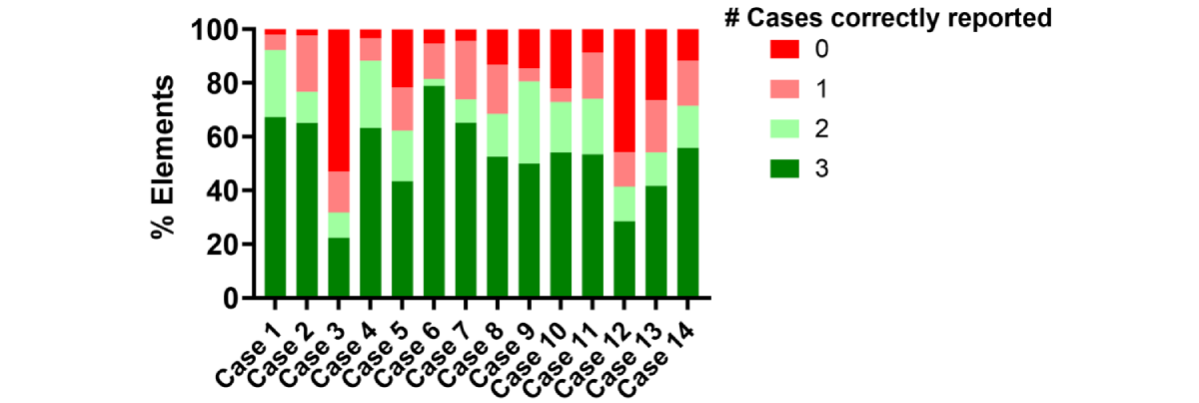
The reproducibility of note accuracy of the ChatGPT-4–generated notes. The percentages of data elements that were reported correctly across 3, 2, 1, or 0 ChatGPT-4–generated replicates were compared across cases.

For accuracy, we assessed the overall congruence between replicates. The frequency of correct reporting across the 3 replicates was compared against the gold standard History and Physical Examination. Overall, the mean percentage of elements reported correctly across all 3 replicates for the 14 cases was 53% (range 22%-79%). Interestingly, nearly 30% of data elements were reported correctly in only 1 or 2 of the replicates, suggesting issues with both accuracy and congruency (Figure 3).

**Figure 4:**
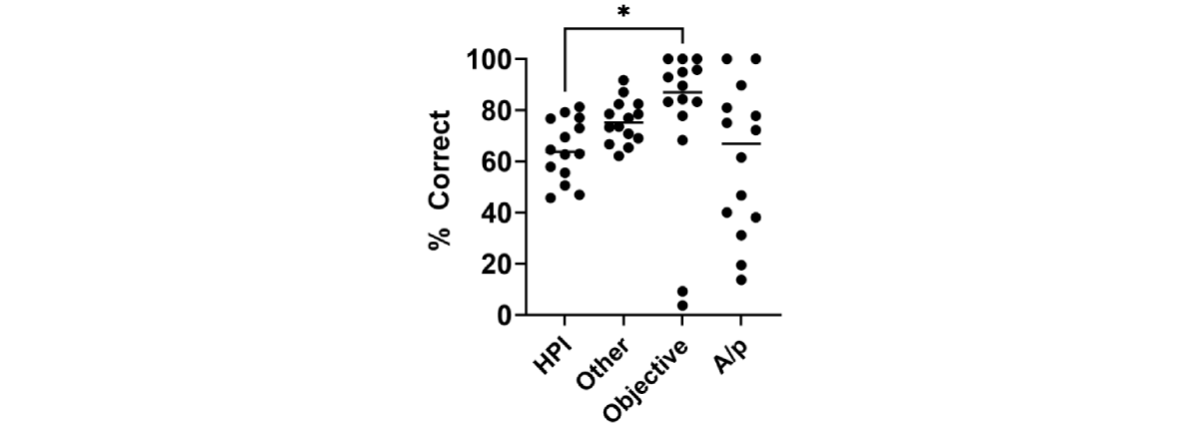
The percentages of correct elements averaged per case based on note category. Each transcript was run through ChatGPT-4 three times and the percentages of correct data elements were averaged across the replicates. The data elements were divided into History of present illness (HPI), Other (eg, medications, allergies, family history, social history, and past medical history), Objective (eg, vital signs, physical exam, and test results), and Assessment and Plan (A/P). The average percentage of correct data in each case was compared based on these documentation categories. The overall difference between groups was significant (*P*=.02). * indicates there was a statistically significant difference between the HPI and the Objective sections (*P*<.05 was considered significant).

Breaking down ChatGPT-4’s performance based on individual categories, there was a significant variance in the accuracy in each section of the note. Specifically, the highest accuracy was observed in the Objective section of the note (median 86.9, IQR 75.4%-96.9%) and was significantly higher compared to the History and Physical examination (median 63.8%, IQR 54.2%-76.8%; *P*=–.02), Other (median 75.2%, IQR 68.5%-82.4%), and Assessment and Plan (median 66.9%, IQR 36.4%-83.5%; Figure 4).

**Figure 5:**
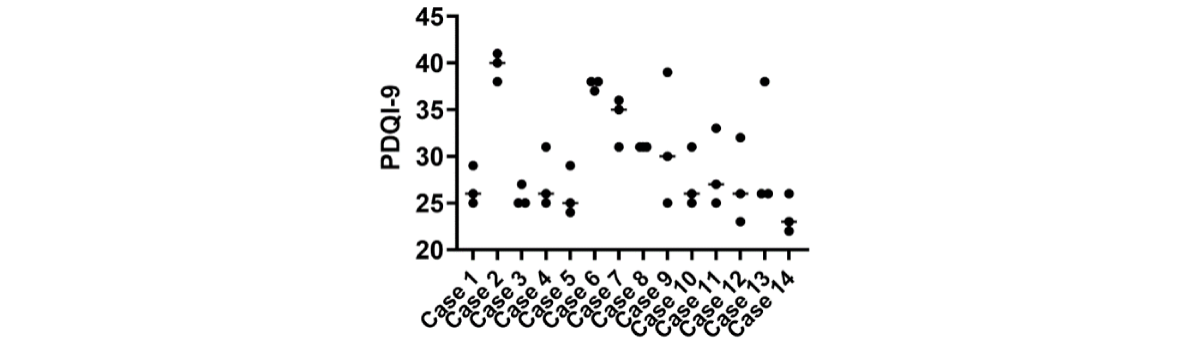
Quality of ChatGPT-4 notes per case. The Physician Documentation Quality Instrument-9 (PDQI-9) scoring system was used to evaluate the quality of the generated notes and then compared across the 14 cases.

The combination of variance in note structure as well as the number and type of errors resulted in similar variance in overall note quality as determined by PDQI-9. Overall, the mean PDQI-9 score was 29.7 (range 23.7-39.7), with significant variance between replicates within a case (Figure 5).

**Figure 6:**
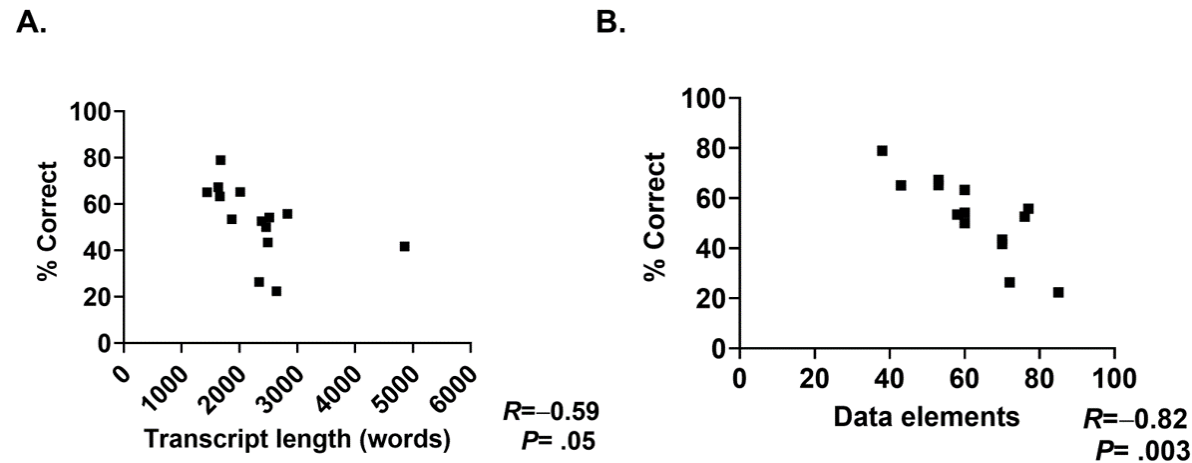
The accuracy of the ChatGPT-4–generated notes. The percentage of correct data elements present in all 3 note replicates was compared against (A) the original transcript length and (B) the number of data elements per case.

Finally, we wished to determine whether characteristics in the parent transcript were associated with note quality. Overall, transcript length and the total number of scorable elements (as a measure of information density or complexity) both correlated inversely with the total percentage of elements reported correctly across the 3 replicates for each case (Figure 6). We observed similar findings for PDQI-9 (details are not shown).

## Discussion

### Principal Results

Our study highlights the significant variations in errors, accuracy, and quality of SOAP notes generated by ChatGPT-4. With regard to errors, they are not limited to specific sections of the note and include errors of omission as well as commission. Although the number of errors is consistent with regard to the number of data elements, another important finding is that the error rate is not consistent across replicates of the same case. This means that the model is not making the same errors repeatedly, making it difficult for health care providers to predict where errors may occur. This variability introduces a level of unpredictability, which can impact clinical oversight.

### Comparison With Prior Work

In the context of medical research, our investigation has shed light on the critical issue of documentation accuracy, which has been a recurring concern in prior studies. Our findings align with the existing body of research on digital scribes, revealing noteworthy variations in accuracy, particularly in the context of nonobjective data [4,14]. In the realm of ChatGPT-4, the study conducted by Johnson et al [15] delved into its performance in giving precise and comprehensive medical information. This inquiry enlisted the expertise of 33 physicians, spanning 17 different specialties, who formulated questions that were subsequently posed to ChatGPT. Approximately 57.8% of the generated responses were assessed as accurate or nearly correct. This outcome underscores the imperative for exercising caution when solely relying on AI-generated medical information and the need for continuous evaluation, as others have noted [16]. However, in another study by Walker et al [17] aimed at evaluating the reliability of medical information provided by ChatGPT-4, multiple iterations of their queries executed through the model yielded a remarkable 100% internal consistency among the generated outputs [17]. Although promising, it should be noted that the queries used in their experiment consisted of direct single-sentence questions pertaining to specific hepatobiliary diagnoses. This mode of input differs significantly from the transcription of patient encounters. Our research, in contrast, stands out by probing the reproducibility of note generation—a relatively less explored topic in existing literature.

The PDQI-9 scores also highlight the overall variance in quality. In previous research, the PDQI-9 score of 26.2 was rated “terrible or bad,” versus a PDQI-9 score of 36.6, which was rated “good or excellent” [13]. In our study, the mean PDQI-9 score of 29.7 is closer to the “terrible or bad” range. These observations suggest that although ChatGPT-4 can consistently generate a SOAP-style note, it introduces errors and struggles with maintaining uniformity and accuracy. These issues could pose potential challenges if implemented in a clinical setting.

An essential aspect of our research was to identify the factors contributing to inaccuracies in AI-generated notes. Notably, we found an inverse correlation between note accuracy and transcript length as well as the amount of reportable data. This observation has profound implications for large language models like ChatGPT-4, indicating their challenges with longer and denser information. This raises questions about their effectiveness in handling complex medical cases.

These findings have significant clinical implications. The high variability in PDQI-9 scores, coupled with a high error rate, indicates low-quality notes. Recently, there have been concerns regarding ChatGPT-4’s capacity to generate what can be classified as “hallucinations”—synthesized data that may be misinterpreted as factual information. These data are often incomplete and sometimes misleading [18]. This has implications for the quality of patient care, potentially leading to diagnostic errors and eroding trust in AI, both among health care providers and patients. Acknowledging the increasing documentation burden contributing to physician burnout, generative AI technology for clinical note documentation may save time [2,19]. However, if our data are representative of similar accuracy rates with other AI-powered systems, any time savings could be negated by the need for corrections. This mirrors previous studies with human scribes, where widespread adoption had little impact on after-hours charting or chart completion time [20-22].

### Limitations

Our research is not without limitations. Primarily, the generated SOAP notes underwent processing through an open AI model, in contrast to the proprietary closed models commonly used in the generative AI domain of health care. It is pertinent to note that proprietary technologies, such as DAX Copilot (a collaborative venture of Microsoft and Nuance), have restricted accessibility, available only to entities holding contractual agreements with the parent company. Furthermore, these models evolve iteratively. Consequently, the errors as well as the correct elements in our current data set might not manifest in subsequent versions. However, it is important to note that the methodology reported here establishes a means by which these systems can be evaluated systematically. It should be acknowledged that this study only used transcripts, eliminating the confounder of any potential errors introduced by the speech recognition aspect [23,24]. Integrating this aspect will be critical for a more complete evaluation of fully integrated generative AI–powered documentation assistants. Another limitation is the inability to draw conclusions regarding the correlation between types of cases and associated errors. A substantially larger volume of encounters would be required to delineate this relationship. Additionally, despite its standardized criteria, using the PDQI can still be influenced by the subjective judgment of the reviewer and can be a time-consuming process, particularly for longer documents. Conversely, the instrument does cover a broad range of quality dimensions, facilitating a more holistic evaluation. Furthermore, it can be used as a diagnostic tool to identify strengths and weaknesses, guiding targeted quality improvement initiatives. Finally, in large language models, such as ChatGPT-4, the temperature of the model is a parameter that controls the randomness or predictability of the model’s output. It is a component that tunes the model to generate responses that are either more varied and creative or more deterministic and conservative. With this in mind, ChatGPT-4’s temperature allows for variability, but this setting is not accessible to the end user [25,26]. Further, even setting the temperature to zero does not appear to ensure uniformity of response [27]. Along these lines, the absence of real-time feedback within the application also limits the model’s ability to adjust its responses based on user input, and therefore, hinders the model’s opportunity to learn from real-world interactions and refine its output.

### Conclusions

In conclusion, our study used standardized simulated patient-provider interactions to evaluate the quality and reliability of AI-generated clinical notes. The generated notes do not meet the acceptable quality standards for clinical use. Our methodology provides a foundation for future assessments of AI technology in terms of quality and safety. At this time, AI should not be considered a safe alternative to digital scribes.

## Appendix

Multimedia Appendix 1 ChatGPT-4 responses.

## Abbreviations

AI: artificial intelligence
LLM: large language model
PDQI: Physician Documentation Quality Instrument
SOAP: Subjective, Objective, Assessment, Plan

## Data Availability

The data sets generated and analyzed during this study are available from the corresponding author on reasonable request.
