# Supplementary material for: Using ChatGPT-4 to Create Structured Medical Notes From Audio Recordings of Physician-Patient Encounters: Comparative Study
Source: J Med Internet Res. 2024 Apr 22;26:e54419. doi: 10.2196/54419 (PMC11074889; doi:10.2196/54419)
Supplement: Multimedia Appendix 1 [file jmir_v26i1e54419_app1.docx]

**ChatGPT- 4 Generated Responses:**

**The Prompt:**

*Generate a clinical note in SOAP format for the following:*

*[insert corresponding cases’ transcript]*

**Case 1: Gastroenteritis**

**Version 1:**

Based on the provided conversation, here's a clinical note in SOAP format for Sacha Diggs:

**S (Subjective):**

- Chief Complaint: Abdominal pain and diarrhea for about three days.

- The pain is cramp-like, all over the abdomen and comes and goes.

- The diarrhea is watery, frequent (up to 10 times daily) and has had blood and mucus in the last two occurrences.

- No vomiting but has decreased appetite due to pain.

- Decreased energy and has noticed dehydration symptoms.

- Eating and drinking exacerbate the pain.

- No similar symptoms noted among contacts/roommates.

- Slightly decreased urinary frequency.

- Recent travel to Argentina for rock climbing.

- Lives with two roommates in North Portland and is a rock climbing instructor.

- No recent sexual activity.

- Allergic to shellfish, which causes abdominal cramping, hives, and throat/chest tightness.

- Family History: Father alive and healthy; mother passed away in a car accident; one sister who is healthy.

**O (Objective):**

- Vital signs: Temp: 101°F, Heart rate: 118 bpm, BP: 108/58 mmHg, RR: 16/min, Height: 5'8'', Weight: 172 lbs.

- Physical Exam:

- Dry skin and oral mucosa.

- Hyperactive bowel sounds.

- Generalized abdominal tenderness without organomegaly or masses.

**A (Assessment):**

- Likely Traveler's Diarrhea with potential infectious etiology given recent travel to Argentina.

- Differential includes infectious causes, exacerbated by dehydration.

**P (Plan):**

- Stool culture to identify potential pathogens.

- Start Sacha on Azithromycin 1 gram, single dose.

- Recommend over-the-counter loperamide (Imodium) for symptom control.

- Emphasize hydration.

- Consideration of probiotics.

- Return precautions provided: If symptoms worsen or new symptoms appear, seek immediate medical care.

- Follow-up once stool culture results are available.

Patient educated on the aforementioned and verbalized understanding. No further questions at this time.

**Version 2:**

**SOAP Clinical Note**

*Patient:* Sacha Diggs

*Date:* [Date of the encounter]

**S - Subjective:**

- Chief Complaint: "My gut’s just messed up."

- Abdominal pain and diarrhea for 3 days.

- Pain is cramping in nature, all over the stomach area, rated at 4/10 in intensity.

- Diarrhea described as watery, with a frequency of about 10 times/day. Noted some blood and mucus in the last two episodes.

- No fever, but feels fatigued.

- Pain exacerbated by eating or drinking.

- Takes Tylenol for pain, with slight relief.

- Fully vaccinated against COVID, not vaccinated for the flu.

- No other medications or supplements.

- No known medical history. Allergic to shellfish (manifests with abdominal cramping, hives, and chest/throat tightness).

- Recently traveled to Argentina for rock climbing.

- Occupation: Rock climbing instructor. Other hobbies include snowboarding, hiking, and surfing.

- Lives in a house in North Portland with two roommates. No one else has similar symptoms.

- No tobacco, occasional weekend alcohol, no drug use, and morning coffee for caffeine. No recent sexual activity.

- Father alive and healthy, mother deceased due to car accident. One healthy sister.

**O - Objective:**

- Vital Signs:

- Temperature: 101°F

- Heart Rate: 118 bpm

- Blood Pressure: 108/58 mmHg

- Respiratory Rate: 16/min

- Height: 5’8’’

- Weight: 172 lbs

- Physical Exam:

- Skin appears dry.

- Oral mucosa dry.

- Abdomen hyperactive with generalized tenderness. No palpable masses. Organs normal in size.

**A - Assessment:**

Possible infectious diarrhea, likely related to recent travel to Argentina. The differential diagnosis includes travelers' diarrhea, potential parasitic infection, or other types of infectious gastroenteritis.

**P - Plan:**

1. Send stool for culture to identify the causative pathogen.

2. Prescribe azithromycin (1 gram, single dose) for suspected bacterial infection.

3. Recommend over-the-counter Imodium to manage diarrhea.

4. Advise on maintaining hydration, possibly with oral rehydration solutions or electrolyte beverages.

5. Suggest considering probiotics for gut flora restoration.

6. Advise the patient to return or seek emergency care if symptoms worsen or new symptoms appear.

7. Follow up on stool culture results and adjust treatment accordingly.

Doctor's Signature: Dr. Collins

Date: [Date of the encounter]

**Version 3:**

**Clinical SOAP Note**

*Subjective*

**Chief Complaint (CC):**

- Patient, Sacha Diggs, presents with a primary complaint of abdominal cramping and diarrhea.

**History of Present Illness (HPI):**

- Patient reports symptoms of abdominal pain and diarrhea for the past three days.

- Describes pain as cramping in nature, which is felt all over the stomach area.

- Diarrhea occurs approximately 10 times a day and is described as watery.

- The last two episodes included blood and mucus.

- Patient denies fever but feels fatigued.

- Ingesting food or drinks exacerbates the pain.

- Taking Tylenol provided minimal relief.

**Past Medical History (PMH):**

- No significant past medical issues.

- Fully vaccinated for COVID but not for the flu.

- Allergic to shellfish with reactions including abdominal cramps, hives, and chest tightness.

**Social History (SH):**

- Lives in a house in North Portland with two roommates.

- Works as a rock climbing instructor.

- Returned recently from a trip to Argentina for rock climbing.

- Enjoys snowboarding, hiking, and surfing.

- Denies tobacco and drug use.

- Consumes coffee and occasional alcohol.

- No recent sexual activity.

**Family History (FH):**

- Father is alive and healthy.

- Mother passed away from a car accident when patient was young.

- Has a healthy sister.

*Objective*

**Vitals:**

- Temperature: 101°F

- Pulse: 118 beats per minute

- Blood Pressure: 108/58 mmHg

- Respiratory Rate: 16 breaths per minute

- Height: 5'8"

- Weight: 172 lbs

**Physical Examination:**

- Skin: Dry

- Oral mucosa: Dry

- Abdomen: Hyperactive bowel sounds. Generalized tenderness upon palpation. No palpable masses or organomegaly.

*Assessment and Plan*

**Assessment:**

- Gastrointestinal infection likely secondary to travel in Argentina.

**Plan:**

1. Obtain a stool culture to identify potential pathogens.

2. Start the patient on a single dose of azithromycin (1 gram).

3. Recommend over-the-counter loperamide (Imodium) for symptom relief.

4. Advise increased fluid intake to compensate for losses from diarrhea.

5. Consider probiotics to help restore gut flora.

6. Follow up with stool culture results and adjust treatment as needed.

7. If symptoms worsen or new symptoms develop, the patient should seek immediate medical attention.

*Documentation completed by the medical scribe.*

**Case 2: Incarcerated Inguinal Hernia**

**Version 1:**

**Clinical Note: SOAP Format**

**Subjective:**

Mr. Cameron Basil, a male patient, presented with a chief complaint of persistent abdominal pain for the past two days. He described the pain as constant. The onset of the pain was shortly after lifting a heavy bag. Mr. Basil denied any prior episodes of such pain. He also reported two episodes of vomiting today and has been constipated. No associated fever, chills, diarrhea, or urinary symptoms. No blood or burning sensation during urination. No allergies were noted. His current medication is Stalevo 100 (25 mg carbidopa, 100 mg levodopa, 200 mg entacapone) for Parkinson's Disease. He does not take any over-the-counter medication. He quit smoking about 20 years ago, doesn't consume alcohol due to his medication, and does not use recreational drugs. Family history is unknown as Mr. Basil is adopted.

**Objective:**

* **Vitals:**

* Temperature: 100.4°F

* Heart Rate: 103 bpm

* Blood Pressure: 146/93 mmHg

* Respiratory Rate: 18 breaths/min

* Oxygen Saturation: 98% on room air

* Weight: 220 pounds

* Height: 6’1” (there was an initial error indicating 5'1")

* **Physical Examination:**

* Abdomen: Tender, especially on the right side.

* Groin: Evident hernia on the right side.

**Assessment:**

Given Mr. Basil's symptoms of persistent abdominal pain, vomiting, constipation, and physical examination findings, there is a high suspicion of an incarcerated or strangulated hernia leading to possible bowel obstruction.

**Plan:**

1. Urgently refer Mr. Basil to the emergency department.

2. Arrange for a CAT scan in the emergency department to evaluate the suspected bowel obstruction.

3. Inform the emergency department about Mr. Basil's condition and the suspicion of bowel obstruction due to hernia.

4. Consider possible surgical intervention if the obstruction is confirmed.

5. Notify the facility where Mr. Basil resides regarding his transfer to the emergency department.

Note: While not explicitly outlined, ensuring continuous monitoring, pain management, and supportive care are essential components of the plan moving forward.

**Version 2:**

**Clinical Note - SOAP Format**

**Subjective**:

*Chief Complaint*: Abdominal pain, persistent for 2 days.

*History of Present Illness*:

- Mr. Cameron Basil, a 6'1" male with a weight of 220 pounds, presented with a 2-day history of constant abdominal pain.

- He mentions onset after lifting a heavy bag.

- Other symptoms include two episodes of vomiting today, constipation, and loss of appetite.

- Denies any fever, chills, diarrhea, or urinary complaints.

- Vomit did not contain any abnormal colors or substances.

- Last intake was coffee at 8 AM. No appetite since.

- Denies recent over-the-counter medications or treatments for the symptoms.

- No previous episodes of similar complaints.

- He is a former smoker, having quit 20 years ago. He does not drink alcohol due to his Parkinson's medication. He denies recreational drug use.

*Past Medical History*:

- Parkinson's disease.

- No other medical conditions.

- Takes Stalevo 100 which contains 25 mg carbidopa, 100 mg levodopa, and 200 mg entacapone for Parkinson's.

*Family History*:

- Adopted. No known family history.

*Social History*:

- No alcohol use. Quit smoking 20 years ago.

- No recreational drug use.

- Children live in California.

**Objective**:

*Vital Signs*:

- Temperature: 100.4°F.

- Heart Rate: 103 bpm.

- Blood Pressure: 146/93 mmHg.

- Respiratory Rate: 18 breaths per minute.

- Oxygen Saturation: 98% on room air.

*Physical Examination*:

- Abdomen: Tenderness on the right side. No obvious lumps or bumps.

- Groin: Presence of hernia on the right side upon coughing.

**Assessment and Plan**:

- *Impression*: Suspected bowel obstruction secondary to right-sided hernia, possibly incarcerated.

- *Plan*:

1. Urgent CT scan evaluation to assess bowel integrity and severity of obstruction.

2. Refer to the emergency department for further evaluation and possible surgery.

3. Notify the patient's facility of the situation.

4. Continue monitoring for worsening symptoms or new-onset complications.

**Plan for Follow-Up**:

- Mr. Basil is to be seen in the emergency department urgently.

- Dr. Chen will meet with the patient in the emergency department and will admit the patient under her service if needed.

**End of Note**.

**Version 3:**

**Clinical Note: Dr. Chen's Consultation with Cameron Basil**

**Subjective:**

**Chief Complaint:**

- Abdominal pain for the past 2 days.

**History of Present Illness:**

- Mr. Cameron Basil (prefers to be called Cameron) reports a constant abdominal pain that has persisted for two days.

- The pain onset after lifting a heavy bag.

- This is a new symptom and has never occurred in the past.

- Accompanying symptoms include: vomiting twice today (contents described as regular vomit, no red or green discoloration), and constipation.

- No reported fever, chills, diarrhea, urinary disturbances, or hematuria.

**Medications:**

- Stalevo 100 for Parkinson's: 25 mg carbidopa, 100 mg levodopa, 200 mg entacapone.

**Past Medical History:**

- Parkinson's disease.

**Past Surgical History:**

- No surgical history. Still has his appendix and gallbladder.

**Family History:**

- Unknown due to adoption.

**Social History:**

- Former smoker (quit 20 years ago).

- Rarely consumed alcohol and abstains currently due to interactions with Parkinson's medication.

- Denies recreational drug use.

**Objective:**

**Vital Signs:**

- Temperature: 100.4°F

- Heart Rate: 103 bpm

- Blood Pressure: 146/93 mmHg

- Respiratory Rate: 18 breaths/min

- Oxygen Saturation: 98% on room air

- Weight: 220 pounds

- Height: 6' 1" (corrected from error stating 5'1")

**Physical Exam:**

- Abdomen: Tender on the right side. No palpable masses.

- Groin: Hernia noted on the right side.

**Assessment and Plan:**

**Diagnosis:**

- Likely right-sided hernia, with the possibility of a bowel blockage causing Cameron's symptoms.

**Plan:**

- Urgent evaluation with a CAT scan is recommended.

- Immediate transfer to the emergency department. An ambulance will be arranged.

- Potential surgical intervention is under consideration.

- Cameron will be admitted under Dr. Chen's service if needed.

**Education and Patient Understanding:**

- The findings and plan were discussed with Cameron, who agrees with the proposed plan of action.

**Follow-up:**

- Dr. Chen will meet with Cameron in the emergency department to further assess and manage his condition.

**End of Note.**

**Case 3: Diabetic Ketoacidosis**

**Version 1:**

**SOAP NOTE**

**S**ubjective:

- **Chief Complaint**:

Hollie Fisher, a young adult, presents with complaints of general malaise, feeling unwell, weakness, and fatigue for the past couple of weeks which intensified in the past few days.

- **History of Present Illness**:

- Onset: Started feeling unwell and weak roughly 2 weeks ago.

- Provocative/Palliative: Nausea and vomiting seem to worsen the pain. Tylenol provided uncertain relief.

- Quality: Described as "achy" abdominal pain, mainly in the upper part.

- Radiation: No indication of pain radiating to other parts.

- Severity: Pain in the abdomen rated 3/10.

- Timing: No specific timing, constant since onset.

- Associated symptoms: Fever, mild cough with some phlegm, and increased frequency of urination for about two weeks which then tapered off.

- Other: Patient arrived by ambulance today.

- **Past Medical History**:

- No known ongoing medical conditions.

- No past surgeries.

- Family history of diabetes (both parents).

- Allergies: None reported.

- Medications: Mentioned Tylenol.

- Social History:

- Student studying art.

- Lives alone in a dormitory.

- Occasional use of edible marijuana.

- No tobacco or alcohol use.

- Consumes a cup of coffee daily.

- **Review of Systems**:

- Fever: Yes.

- Nausea/Vomiting: Yes.

- Abdominal pain: Yes, upper part.

- Change in urination: Increased frequency initially, decreased after vomiting started.

- Cough: Mild, with some phlegm.

**O**bjective:

*(Note: Exam results not provided in the given conversation, but a typical examination would cover general appearance, vital signs, physical findings, etc.)*

- **General**: Hollie appears distressed.

- **Vital Signs**: Not provided.

- **HEENT**: Not provided.

- **Lungs**: Not provided.

- **Cardiac**: Not provided.

- **Abdominal**: Point tenderness in the upper part.

- **Neurologic**: Alert and oriented x3.

**A**ssessment:

- Differential Diagnoses:

1. Viral gastroenteritis.

2. Acute pancreatitis.

3. Pyelonephritis.

4. Gallbladder pathology.

5. Possible early signs of diabetes, given family history.

**P**lan:

- **Investigations**:

1. Complete Blood Count (CBC).

2. Basic Metabolic Panel (BMP) to check kidney function and glucose levels.

3. Liver function tests.

4. Urinalysis.

5. Possible imaging (e.g., ultrasound) to assess for gallbladder pathology.

- **Treatment**:

1. Maintain hydration and monitor fluids due to vomiting.

2. Pain management as needed, considering her mild pain.

3. Monitor and manage fever.

- **Education**:

1. Discuss the importance of maintaining hydration, especially after vomiting.

2. Advise Hollie to avoid edibles and any other drugs until diagnosis is clear.

3. Reinforce the importance of timely follow-up and when to seek emergency care (e.g., increased pain, inability to keep fluids down).

- **Follow-up**:

Based on test results and response to initial management, further appointments or referrals might be needed.

*(Note: The plan would be adjusted based on actual exam findings and results from diagnostic tests.)*

**Version 2:**

**Clinical Note (SOAP Format)**

**Subjective:**

*Chief Complaint:* "Feeling really unwell" for the past couple of weeks.

*History of Present Illness:* Hollie Fisher, a college student studying art, presented with a 2-week history of generalized weakness and fatigue, which worsened over the past few days. She reported a fever and upper abdominal pain (described as "achy") that began three days ago. Over the past two days, she developed nausea and vomiting. She noted increased frequency of urination over the past two weeks, which decreased after the vomiting started. She also has a mild cough and has produced a small amount of phlegm. No known sick contacts, no recent dietary changes. No recent travel or medication use. No known history of diabetes or hypertension. She is sexually active, with her last menstrual period about three weeks ago and reported regular cycles.

*Past Medical History:* No known ongoing medical conditions. No prior surgeries.

*Family History:* Father diagnosed with diabetes in childhood, mother may also have diabetes.

*Social History:* Currently in college studying art. Lives in a dorm alone. Uses cannabis edibles occasionally. No tobacco or alcohol use. Drinks a cup of coffee daily.

**Objective:**

*General:* Alert, oriented, and in no acute distress.

*Abdominal:* Mild tenderness in the upper quadrant, with no guarding or rebound. No hepatosplenomegaly.

*Respiratory:* Mild cough with a small amount of expectoration. No wheezing or crackles noted.

*Other Systems:* Detailed physical examination findings pending.

**Assessment and Plan:**

1. **Generalized weakness and fatigue:** The differential includes a variety of conditions from infectious causes to metabolic disturbances. Given the associated symptoms, it will be essential to rule out conditions like mononucleosis, diabetes (especially with the family history), thyroid dysfunction, anemia, etc.

2. **Upper Abdominal Pain and Nausea/Vomiting:** The differential includes gastritis, peptic ulcer disease, gallbladder pathology, or pancreatitis. Would consider ordering a basic metabolic panel, liver function tests, lipase/amylase, and a right upper quadrant ultrasound.

3. **Increased Urination:** Given the family history of diabetes, it is imperative to check for undiagnosed diabetes. Will order fasting blood glucose and Hemoglobin A1c.

4. **Cough:** Although described as mild, with the context of recent illness, it may be worth considering a chest x-ray and perhaps respiratory viral panels.

Overall, based on the physical examination findings, further workup with blood tests, imaging, and potential specialist referrals will be considered.

**Plan:**

- Order labs including CBC, BMP, LFTs, lipase, amylase, fasting glucose, HbA1c.

- Consider ordering a right upper quadrant ultrasound to evaluate the liver, gallbladder, and pancreas.

- Chest X-ray to rule out any respiratory pathology.

- Provide symptomatic relief for nausea and pain. Monitor vital signs closely.

- Consider admitting for observation and further workup if results are abnormal or if symptoms worsen.

End of note.

**Version 3:**

**CLINICAL NOTE - SOAP FORMAT**

**S: Subjective**

*Chief Complaint:*

Patient Hollie Fisher presents stating, "I’ve just been feeling really unwell and just, like, not good for the past couple weeks."

*History of Present Illness:*

For the past couple of weeks, Ms. Fisher has been feeling weak and fatigued. This was exacerbated three days ago when she started having a fever and abdominal pain localized to her upper abdomen, described as achy. One day after the onset of the fever, she started feeling nauseous and has been vomiting since. She rates her pain as 3 out of 10. She also has a mild cough and has been bringing up a small amount of phlegm. No significant dietary changes noted. There's a significant change in urination habits - increased frequency of urination in the past two weeks which reduced around the time she started vomiting.

*Past Medical History:*

No known ongoing medical conditions or surgeries.

*Family History:*

Father and possibly mother have diabetes, likely Type I as her father had it since childhood.

*Social History:*

Ms. Fisher is an art student living in a dorm. She occasionally uses cannabis edibles but does not use tobacco or alcohol. She consumes a cup of coffee daily.

**O: Objective**

*Vital Signs:*

Not provided.

*Physical Examination:*

- General: Appears ill.

- Abdomen: Localized pain in the upper quadrant. (Further findings pending physical examination)

- Respiratory: Mild cough with minimal sputum production. (Further findings pending physical examination)

**A: Assessment**

Differential Diagnoses:

1. Viral gastroenteritis

2. Acute gastritis

3. Gallbladder disease (e.g., cholecystitis or gallstones)

4. Pancreatitis

5. Possible urinary infection given the change in urination habits

6. Possible early stages of diabetes mellitus, considering the family history and increased frequency of urination.

**P: Plan**

1. **Laboratory Tests:**

- Complete Blood Count (CBC) to check for signs of infection

- Basic Metabolic Panel (BMP) to assess electrolytes, kidney function, and blood sugar levels

- Liver Function Tests (LFTs)

- Lipase and amylase levels to evaluate for pancreatitis

- Urinalysis to assess for UTI or other renal conditions

2. **Imaging:**

- Abdominal ultrasound to evaluate gallbladder and pancreas

3. **Treatments:**

- Provide IV fluids for hydration due to nausea and vomiting

- Consider anti-emetic medication to control vomiting

- Pain management as required

4. **Follow-up:**

- Monitor vitals and clinical symptoms

- Review laboratory and imaging results

- Discuss findings and further management with Ms. Fisher.

This is a mock SOAP note based on the provided patient-physician interaction. Clinical judgment and evaluation may reveal other considerations or diagnoses.

**Case 4: Ovarian Cyst**

**Version 1:**

**Clinical Note** - **SOAP Format**

**Subjective:**

**Chief Complaint:**

Abdominal pain for two days, associated with the onset of menstrual cycle.

**History of Present Illness (HPI):**

- Naomi Parks, presents with a complaint of abdominal pain localized to the lower right quadrant of her abdomen.

- The pain began approximately two days ago, coinciding with the onset of her menstrual cycle.

- Describes the pain as sharp and intermittent.

- Reports one episode of vomiting a clear yellow fluid.

- Reports increased frequency of urination but denies dysuria or hematuria.

- Denies any changes in bowel habits.

- Reports recent irregularities in her menstrual cycle with increased flow.

- Reports no chance of current pregnancy and denies prior pregnancies.

- Reports use of ibuprofen for pain with little relief.

- Past medical history is insignificant.

- Family history significant for ovarian cysts in mother and aunt.

**Medications:**

- Occasionally uses laxatives.

- Has taken ibuprofen for pain.

**Allergies:**

- Amoxicillin, which caused a rash as an infant.

**Social History:**

- Denies tobacco use, has never used.

- Consumes wine occasionally, approximately a few times a week.

- Denies use of recreational drugs.

- Not on birth control.

**Objective:**

**Vitals:**

- Temp: 97.6°F

- HR: 110 bpm

- BP: 138/88 mmHg

- RR: 19/min

- SpO2: 98%

- Weight: 135 lbs

**Physical Examination:**

- Patient in mild distress.

- No anemia or scleral icterus noted.

- Abdomen soft, non-distended with minimal guarding.

- Tenderness present in the right iliac fossa. No rebound tenderness.

- Bowel sounds active.

**Assessment & Plan:**

**Assessment:**

- Differential diagnosis includes ovarian cyst, appendicitis, kidney stones, and urinary tract infection based on presentation and family history.

**Plan:**

- Obtain labs including CBC and CMP.

- Urinalysis to rule out infection and check for blood.

- Urine pregnancy test to rule out pregnancy.

- CT scan of the abdomen to provide a clear view of the pathology.

- Once the cause is determined, consultation with the appropriate specialty will be pursued.

**Discussion with Patient:**

Explained potential causes of her symptoms and the necessary tests to determine the cause. Emphasized the possibility of an ovarian cyst but stated that other conditions, such as appendicitis and kidney stones, need to be ruled out. The patient appeared to understand and did not have any further questions at this time.

**Follow-up:**

Will review results of the investigations once they are available and discuss further management options with the patient.

**Version 2:**

Clinical Note (SOAP format):

**Subjective:**

Naomi Parks, a young female, presents with a chief complaint of abdominal pain which has been ongoing for the past two days. She describes the pain as a sharp sensation located in the lower right abdomen that comes and goes. The onset of pain coincided with the start of her menstrual cycle. Ms. Parks also reports an increased frequency in urination but denies any pain during urination or the presence of blood in the urine. Bowel movements appear normal to her. She has vomited once, bringing up yellow-clear fluid with no presence of blood. She has been experiencing irregular and heavier menstrual cycles lately but denies the possibility of being pregnant. She has been taking ibuprofen for the pain without significant relief. Past medical history is notable for a rash reaction to amoxicillin as an infant. Family history reveals the presence of ovarian cysts in both her mother and her aunt.

**Objective:**

* **Vitals:**

- Temperature: 97.6°F

- Heart rate: 110 bpm

- Blood pressure: 138/88 mmHg

- Respiratory rate: 19 breaths/min

- Oxygen saturation: 98% on room air

- Weight: 135 pounds

* **Physical Examination:**

- General Appearance: Patient in mild discomfort

- Eyes: No anemia or scleral icterus

- Abdomen: Soft, non-distended, minimal guarding. Tenderness in right iliac fossa with no rebound tenderness. Bowel sounds normal.

**Assessment:**

The differential diagnosis includes:

- Ovarian cyst: Given the relationship of pain with her menstrual cycle and family history.

- Appendicitis: Due to the location and nature of the pain.

- Kidney stones: Given the increased frequency of urination and the location of pain.

**Plan:**

1. Administer IV pain relief and anti-nausea medication.

2. Bloodwork: CBC and CMP to evaluate for signs of infection and general health status.

3. Urinalysis: To check for signs of infection and presence of blood.

4. Pregnancy test: To rule out ectopic pregnancy or other pregnancy-related conditions.

5. CT scan of the abdomen: To definitively diagnose the cause of her symptoms.

6. Once results are available, consultation with appropriate specialty (e.g., Gynecology if an ovarian cyst is found) will be done to determine the best course of action.

The patient was counseled on the potential diagnoses and plan. She had no further questions at this time and consented to the proposed plan. The importance of following up on the results was stressed. She was advised to seek immediate medical attention if her symptoms worsened.

End of Note.

**Version 3:**

**Clinical SOAP Note:**

**Subjective:**

*Chief Complaint:*

Abdominal pain.

*History of Present Illness:*

Ms. Naomi Parks presents with 2-day history of lower right abdominal pain. She reports the pain is sharp in nature, intermittent, and is localized to the right lower quadrant. The onset of pain coincided with the beginning of her menstrual cycle. No radiation of the pain. Ms. Parks also reports increased frequency of urination. No dysuria or hematuria. She vomited once, bringing up a yellow-clear fluid, with no blood. She has been using ibuprofen with minimal relief. No history of recent trauma, surgeries, or other illnesses.

*Past Medical History:*

No significant medical history.

*Medications:*

Intermittent use of ibuprofen and laxatives.

*Allergies:*

Amoxicillin - caused a rash as an infant.

*Family History:*

Mother and aunt diagnosed with ovarian cysts. No other significant family medical history.

*Social History:*

Tobacco: Never used. Alcohol: Occasionally consumes wine, a glass or few times per week. Recreational drugs: None.

*Review of Systems:*

Menstrual cycles have been irregular recently and heavier than usual. No history of pregnancy. No use of birth control.

**Objective:**

*Vitals:*

- Temperature: 97.6°F

- Heart Rate: 110 bpm

- Blood Pressure: 138/88 mmHg

- Respiratory Rate: 19 breaths/minute

- Oxygen Saturation: 98% on room air

- Weight: 135 lbs

*Physical Examination:*

- General: Alert, oriented, appears in mild discomfort.

- Abdominal: Soft, non-distended. Tenderness in the right iliac fossa with mild guarding. No rebound tenderness. No palpable masses or ascites. Bowel sounds normal.

**Assessment & Plan:**

*Assessment:*

Ms. Parks's presentation of right lower quadrant pain, with a family history of ovarian cysts, and the timing with her menstrual cycle raises the suspicion of an ovarian cyst. However, differential diagnoses to consider include appendicitis and renal stones.

*Plan:*

1. Start IV access.

2. Administer analgesics and anti-emetics.

3. Order laboratory tests: CBC, CMP, Urinalysis, and Pregnancy test.

4. Order imaging: Abdominal CT scan.

5. Re-evaluate after receiving lab and imaging results.

6. Consider consultation with Gynecology if ovarian cyst is confirmed.

*Education & Counseling:*

Educated patient about the potential diagnosis and the need for further tests to confirm. Reassured patient that her condition is treatable, and the medical team will take necessary steps based on results.

*Follow-up:*

Plan to follow-up with Ms. Parks after results of investigations are available.

**Note:**

This is a synthesized SOAP note based on the dialogue provided. In an actual clinical setting, documentation should be detailed, precise, and based on actual patient data and findings.

**Case 5: Pneumonia**

**Version 1:**

**Clinical Note (SOAP Format)**

**Subjective**:

Mr. Lohan, a male patient in the rehabilitation center for the past month, presents to the ED with a complaint of a progressively worsening cough for the past three days. The cough was initially dry but as of this morning has started producing dark yellow sputum. He has been feeling hot with chills and was informed of a fever by the rehab center, although the exact value is unknown. He denies night sweats. Concurrently, he has been experiencing left-sided chest pain exacerbated by deep breaths and coughing. He also reports a history of low back pain, worsening since his knee operation. His sleep has been disturbed due to the cough but denies episodes of gasping for air. He reports feeling short of breath for the same duration of three days. Denies any sickness contact at the rehab center and any current swelling, abdominal complaints, vomiting, joint aches, or muscle pain. However, he feels fatigued, which started around the same time as the other symptoms. He has a known history of hypertension for which he is on Hydrochlorothiazide (HCTZ), taken once daily. He is also taking oxycodone for pain post-knee surgery. Has a past smoking history, with cessation about 20 years ago. Denies alcohol or drug use. Family history includes a father who passed away young (in his early 50s) with unknown cause and a mother who lived to 93. His only surgery is a knee replacement. He currently requires two pillows for sleeping, a change from his usual one, due to his symptoms.

**Objective**:

Vitals:

- BP: 103/43

- HR: 111

- Temp: 38.3 Celsius

- RR: 24

- Weight: 245 pounds

- O2 Sat: 87% on room air; 98% on supplemental oxygen.

Physical Exam:

- Patient appears in mild distress.

- Breath sounds: coarse overall with rhonchi in the left lower lobe.

- Heart sounds: elevated rate but regular without murmurs, rubs, or gallops.

- Abdomen: soft, nontender, nondistended.

- Extremities: Left knee post-surgery, not tender on examination.

**Assessment/Plan**:

1. **Acute Bronchitis/Pneumonia**:

- The presence of productive cough, fever, and rhonchi on examination suggests possible bronchitis or pneumonia. Chest X-ray and sputum culture will be needed for further evaluation.

- Start empirical antibiotics pending culture results.

2. **Chest Pain**:

- Pleuritic nature. Consider Chest X-ray and EKG to rule out pulmonary conditions and cardiac causes.

3. **Shortness of Breath**:

- Possibly related to the respiratory infection. Continue monitoring O2 sats and consider PFTs if symptoms persist.

4. **Hypertension**:

- Monitor blood pressure closely. Consider dosage adjustment of HCTZ if BP remains low.

5. **History of Knee Surgery**:

- Monitor for any signs of post-op complications, given the patient's recent procedure.

6. **Smoking History**:

- Advise on continued cessation and consider a lung function test given the history of smoking.

Patient education and counseling were provided, and Mr. Lohan was encouraged to return if symptoms worsened or new symptoms developed.

**Plan**:

- Order chest X-ray, sputum culture, and EKG.

- Begin empirical antibiotics.

- Monitor vital signs closely.

- Follow up in 48 hours for results and symptom check.

(Note: This is a hypothetical clinical note based on the information provided. In a real-world setting, further diagnostics, interventions, and thorough evaluation would be required.)

**Version 2:**

**SOAP Note:**

**Subjective:**

Mr. Lohan, a patient from a rehabilitation center, presented to the Emergency Department (ED) with a chief complaint of cough that began 3 days ago. The cough was initially dry, but as of this morning, he began producing dark yellow phlegm. He denies any prior episodes of similar symptoms. He also reports fever, shortness of breath that started concurrently with the cough, and having to sleep with two pillows, a change from his usual one pillow. Mr. Lohan has pain in the left side of his chest that he says is exacerbated with deep breaths or coughing. He has a history of low back pain, which he initially thought was related to his knee, for which he had surgery. He denies any other symptoms, including leg swelling, GI symptoms, joint pain, or muscle aches. However, he feels very fatigued.

Past medical history: High blood pressure.

Medications: Hydrochlorothiazide (HCTZ) and oxycodone.

Allergies: Unknown medication that caused a rash and itching post-knee surgery.

Social history: Former smoker, quit 20 years ago. Consumed 10-20 cigarettes/day. Denies alcohol and drug use. Currently residing in a rehab facility for about a month following his knee surgery.

**Objective:**

*Vital Signs*:

BP: 103/43 mmHg

HR: 111 bpm

Temp: 38.3°C

RR: 24/min

Weight: 245 lbs

O2 Sat: 87% on room air, 98% on supplemental oxygen.

Physical Exam:

- Appearance: Mild distress, able to maintain a conversation.

- Pulmonary: Coarse breath sounds with rhonchi noted, predominantly in the left lower lobe.

- Cardiac: Regular rate and rhythm, no murmurs, rubs, or gallops.

- Abdomen: Soft, non-tender, and non-distended.

- Musculoskeletal: Recent left knee surgery, non-tender to palpation.

- No peripheral edema noted.

**Assessment and Plan:**

1. **Respiratory Distress with productive cough**: Given the patient's presentation and physical exam findings, differential diagnosis includes pneumonia, bronchitis, congestive heart failure, or other respiratory infections.

- Plan: Chest X-ray, CBC, Blood cultures, sputum culture, and start on empiric antibiotics. Monitor O2 saturations and consider increasing supplemental oxygen if required.

2. **Chest Pain with deep breaths**: Differential diagnosis includes pleuritis, costochondritis, or cardiac etiology.

- Plan: EKG and cardiac enzymes.

3. **Hypertension**: Continue Hydrochlorothiazide (HCTZ). Monitor BP closely in the ED due to low reading on presentation.

4. **Pain Management**: Given that Mr. Lohan is taking oxycodone for pain, a review of his pain control regimen is needed. The low back pain he reports will also need assessment.

5. **Potential Medication Allergy**: Will need to further investigate the unknown medication that caused the rash during his prior hospitalization.

The patient should be kept for observation, and if he remains stable, a decision on admission versus discharge will be made based on test results and overall clinical picture. If discharged, Mr. Lohan should follow up with his primary care provider and be advised to return to the ED if symptoms worsen.

**Version 3:**

**SOAP NOTE**

**S (Subjective):**

* Chief Complaint: Persistent cough, shortness of breath, chest pain, and low back pain.

* HPI (History of Present Illness): 3-day history of worsening cough initially dry, now productive with dark yellow sputum. Onset of shortness of breath 3 days ago. Wakes up due to cough but not gasping for air. Pain in the left side of chest with deep breaths and cough. No other associated chest pain. Low back pain since before knee surgery. Overall feeling of fatigue since 3 days.

* Past Medical History: Hypertension on hydrochlorothiazide. Knee replacement surgery.

* Medications: Hydrochlorothiazide, oxycodone for knee and back pain.

* Allergies: Possible rash from unknown medication during knee surgery.

* Social History: Former smoker (quit 20 years ago, smoked 10-20 cigarettes/day). No alcohol or drug use. Currently staying in a rehab center for a month after knee replacement surgery.

* Family History: Mother deceased at 93. Father deceased at early 50s, cause unknown.

**O (Objective):**

* Vitals: BP 103/43 mmHg, HR 111 bpm, Temp 38.3°C, RR 24, Weight 245 lbs, O2 sat 87% on room air and 98% on supplemental O2.

* Physical Exam:

* General: Mild distress, able to converse.

* Chest: Rhonchi in left lower lobe, coarse breath sounds.

* Cardiovascular: Tachycardic but regular, no murmurs.

* Abdomen: Soft, non-tender, non-distended.

* Extremities: Left knee post-surgery with no tenderness. No leg swelling.

**A (Assessment):**

* Acute bronchitis or pneumonia with associated hypoxemia.

* Left chest wall pain likely pleuritic.

* Chronic low back pain.

* Hypertension.

* History of left knee replacement.

**P (Plan):**

* Obtain chest X-ray to rule out pneumonia.

* Begin antibiotics targeting common respiratory pathogens.

* Monitor oxygen levels and titrate supplemental oxygen as needed.

* Pain management for chest and back pain.

* Address elevated heart rate - consider fluid resuscitation given low BP.

* Further investigation into the cause of hypoxemia.

* Counseling on importance of deep breathing exercises to improve lung expansion.

* Follow up on rehab center environment for potential causes of symptoms.

**Case 6: Menstrual migraine**

**Version 1:**

**Clinical SOAP Note**

**Subjective**

*Chief Complaint*: Chronic headaches that become severe during her menstrual cycle.

*History of Present Illness*: Yvette Jefferson reports a long history of headaches since childhood but mentions they have been progressively getting worse. She notices a pattern of their onset around the time her period starts, but they are infrequent in between menstrual cycles. The headaches are characterized by a severity level of 7 out of 10, no relief with over-the-counter meds like Tylenol, Motrin, or Excedrin. The pain typically resides on the right side of the head and is accompanied by sensitivity to light and noise, as well as nausea. The headaches last approximately 6-8 hours, during which she usually needs to be in a quiet, dark room.

*Past Medical History*: Diagnosed with Premenstrual Dysphoric Disorder. On sertraline for treatment, which has improved several associated symptoms.

*Medications*: Sertraline, OTC pain medications (Tylenol, Motrin, Excedrin), occasional caffeine for headaches.

*Family History*: Mother experienced headaches and had a history of depression during Yvette’s teenage years.

*Social History*: Non-smoker, consumes approximately four alcoholic beverages per week, no recreational drug use, works as a hairdresser.

**Objective**

*Vital Signs*:

- Temperature: 96.4°F

- Heart rate: 88 bpm (regular)

- Blood Pressure: 118/70 mmHg

- Respiratory Rate: 16 breaths/min

- Weight: 139 pounds

*Physical Examination*:

- Mental status: Normal

- Neurological Exam: Normal

- Cranial nerves: Normal

- Motor Exam: Normal

- Sensory Exam: Normal

- Coordination, Reflexes, Gait: Normal

**Assessment**

Menstrual migraines: Yvette's symptoms, which are severe headaches correlated with her menstrual cycle, match the typical characteristics of menstrual migraines.

**Plan**

- **Medication**: Prescribe Reglan (metoclopramide) to be taken at the onset of the headache or when a headache is anticipated, along with either Tylenol or Motrin. This antiemetic should help with the associated nausea.

- **Lifestyle**: Continue with the headache diary to keep track of headaches, potential triggers, and any other related symptoms.

- **Education**: Send patient education materials through the patient portal about managing migraines and symptom relief.

- **Follow-up**: Schedule a follow-up visit or communicate if the current plan is not effective. A referral to a neurologist or headache center can be considered if the situation doesn’t improve or worsens.

*End of SOAP Note*

**Version 2:**

**Clinical Note - SOAP Format**

**Subjective (S):**

**Chief Complaint (CC):**

- Patient presents with long-term complaints of severe headaches.

**History of Present Illness (HPI):**

- Yvette has experienced headaches since childhood.

- Severity has recently increased, with pain scoring around 7 out of 10.

- Headaches appear to be associated with the onset of her menstrual cycle but are infrequent between cycles.

- Associated symptoms include sensitivity to light and noise, nausea, and a feeling of needing to retreat to a quiet, dark room.

- Duration of headaches ranges from 6-8 hours.

- Pain is localized to the right side of the head.

- OTC medications like Tylenol, Motrin, and Excedrin do not provide substantial relief. Has also tried caffeine.

**Past Medical History (PMH):**

- Premenstrual dysphoric disorder, for which she takes sertraline. Other symptoms like irritability, fatigue, difficulty sleeping, nausea, and bloating have mostly improved with sertraline.

**Family Medical History (FMH):**

- Mother has a history of headaches and experienced depression during Yvette's teenage years.

**Social History (SH):**

- Non-smoker.

- Alcohol: about four glasses per week.

- No recreational drugs.

- Works as a hairdresser and finds her job enjoyable and not stressful.

**Objective (O):**

**Vital Signs:**

- Temperature: 96.4°F

- Heart rate: 88 bpm, regular

- Blood pressure: 118/70 mmHg

- Respiratory rate: 16 breaths/min

- Weight: 139 lbs

**Physical Examination:**

- Neurological exam: Mental status, cranial nerves, motor exam, sensory exam, coordination, reflexes, and gait all normal.

**Assessment (A):**

- Menstrual migraine: Headaches appear to be related to her menstrual cycle and are characterized by moderate to severe pain, sensitivity to light and noise, and nausea. The physical exam and her medical history support this diagnosis.

**Plan (P):**

- **Medication:** Prescribe Reglan (metoclopramide) to be taken during the onset of a headache. Can be combined with OTC pain relievers like Tylenol or Motrin.

- **Lifestyle Modification:** Continue and possibly expand headache diary to track potential triggers or patterns. Patient should note things like caffeine intake, stress, and other potential factors.

- **Education:** Send patient materials on migraine management and symptom relief through the patient portal.

- **Follow-up:** Monitor response to treatment. If there's no improvement or if symptoms worsen, consider referring to a neurologist or headache center.

**Version 3:**

**Clinical SOAP Note**

**Subjective:**

**Patient Demographics:**

Yvette Jefferson, Female.

**Chief Complaint:**

Severe headaches.

**History of Present Illness:**

Patient reports having had headaches since childhood, but recently, they have been more severe. She sometimes senses them coming on, especially around the time of her menstrual cycle. The headaches are less frequent between periods. Patient rates the headache pain as 7 out of 10. When she takes OTC medications like Tylenol or Motrin, she does not notice significant relief. Along with the headache, she experiences sensitivity to light and noise, nausea, and needs to retreat to a dark room. Headaches last approximately 6 to 8 hours. The pain is localized to the right side of her head. No visual disturbances or other neurological symptoms are noted. Patient has been trying to maintain a headache diary.

**Medications:**

- Sertraline

- Occasionally, Tylenol, Motrin, Excedrin, and caffeine for headaches.

**Past Medical History:**

- Premenstrual dysphoric disorder (treated with sertraline; other symptoms like irritability, sleep disturbances, nausea, and bloating have improved with this treatment).

**Family Medical History:**

Mother had headaches and experienced depression during patient’s teenage years.

**Social History:**

- Non-smoker

- Consumes approximately 4 alcoholic drinks per week

- No recreational drug use

- Limited caffeine intake

- Works as a hairdresser, does not find it stressful.

**Objective:**

**Vital Signs:**

- Temperature: 96.4°F

- Heart Rate: 88 bpm (regular)

- Blood Pressure: 118/70 mmHg

- Respiratory Rate: 16 breaths/min

- Weight: 139 pounds

**Neurological Examination:**

- Mental status: Normal

- Cranial nerves: Normal

- Motor exam: Normal

- Sensory exam: Normal

- Coordination, reflexes, and gait: Normal

**Assessment:**

Based on the history and examination, the patient is likely suffering from menstrual migraines, which are migraines that are associated with her menstrual cycle.

**Plan:**

- Prescription: Reglan (metoclopramide) as an antiemetic to help alleviate symptoms associated with the headache.

- Continue to maintain and review headache diary.

- Patient education materials on managing migraines will be sent via the patient portal.

- If symptoms do not improve or worsen after a couple of menstrual cycles, consider referral to a headache center or neurologist.

**Notes:**

The patient was advised about the medication, its side effects, and the importance of adhering to the treatment plan. The patient expressed hope for improvement in her symptoms. Further follow-up will be determined based on the progression of symptoms or any new developments.

**Case 7: Breast Mass**

**Version 1:**

**Clinical Note - SOAP Format**

**Subjective:**

- Chief Complaint: Abnormal nipple discharge and a palpable breast lump in the right breast.

- History of Present Illness:

- Darby Washington, a 5'6", 135-pound female, presents with a two-week history of dark brown to black nipple discharge from her right breast. She noticed a lump in the same breast a couple of months ago. She underwent a mammogram and ultrasound at that time, which were both reassuring and showed benign findings. Her menstrual periods have become irregular, and she has missed her period for the past two months. Darby also reports some unintentional weight loss in the past couple of weeks and fatigue.

- Past Medical History:

- No known medical conditions.

- Over-the-counter medications: Tylenol and Advil as needed.

- Smoker for approximately 15-20 years, half a pack per day but quit 10 years ago.

- Rare alcohol intake, no recreational drug use.

- Family History:

- Father: Deceased from a stroke in his early 80s.

- Mother: Deceased from a housefire five years ago, but reportedly healthy prior.

- Brother: Healthy.

- No known family history of breast cancer.

- Social History:

- Lives with her husband.

- Has one son.

**Objective:**

- Vital Signs:

- Temperature: 97.6°F

- Pulse: 101 bpm

- Blood Pressure: 138/92 mmHg

- Respiratory Rate: 19 bpm

- Oxygen Saturation: 98% on room air

- Physical Examination:

- Breast: Extra glandularity noted in the upper and outer quadrant of the right breast, no other firm mass palpated. Black discharge elicited from the right nipple. No skin abnormalities, such as puckering or "orange peel" appearance. No nipple inversion.

- Lymph nodes: No palpable lymphadenopathy.

- Left breast: Normal examination.

**Assessment:**

- Dark brown to black nipple discharge from the right breast.

- Previously identified breast lump, deemed benign on prior imaging.

- Unintentional weight loss and fatigue.

**Plan:**

- Order an urgent MRI of the right breast for a detailed assessment given the new symptoms. Aim to get the MRI done within a week.

- Encourage Darby to sign up for the patient portal for easy communication regarding her test results and follow-up.

- Based on MRI results, decide on further management and possible biopsy.

*Note: As with any clinical note, this document would be stored securely and maintained confidentially in accordance with medical record-keeping standards and regulations.*

**Version 2:**

**SOAP Note**

**S** (Subjective):

**Chief Complaint:**

Miss Darby Washington presents with a 2-week history of dark brown to black nipple discharge from her right breast, in addition to a lump felt on the same side that was detected months prior.

**History of Present Illness:**

The lump was evaluated on her last annual check-up, and an ultrasound deemed it likely benign. A mammogram was also conducted, which did not show concerning signs. The nipple discharge is more pronounced with compression, but some occurs spontaneously.

**Past Medical History:**

No diagnosed breast conditions or other medical conditions.

**Medications:**

Occasional over-the-counter Tylenol and Advil.

**Social History:**

Smoked half a pack of cigarettes daily for around 15-20 years, but quit 10 years ago on her son's request. Drinks alcohol infrequently (a few times a year). No recreational drug use. Lives with her husband.

**Family History:**

Father died from a stroke in his early 80s. Mother died in a housefire 5 years ago but was reportedly healthy. She has a healthy brother. No known family history of breast cancer.

**Review of Systems:**

- Changes in menstruation for the past 6 months; it's been almost 2 months since her last period.

- No hormone replacement therapies or hormonal contraceptives used.

- Fatigue.

- Slight unintentional weight loss in the last two weeks.

- No fevers or chills.

**O** (Objective):

**Vitals:**

Temperature: 97.6°F, Heart Rate: 101 bpm, Blood Pressure: 138/92 mmHg, Respiratory Rate: 19 bpm, Oxygen Saturation: 98% on room air, Weight: 135 lbs, Height: 5'6''

**Physical Exam:**

On examination, increased glandularity was noted in the upper outer quadrant of the right breast. No other palpable masses, no skin changes, no nipple inversion, no enlarged lymph nodes. Discharge of dark color can be elicited from the right nipple. The left breast appeared normal.

**A** (Assessment):

1. Dark nipple discharge from right breast of unknown etiology.

2. Lump previously noted on the right breast, deemed likely benign based on past ultrasound.

3. Fatigue and slight unintentional weight loss, cause undetermined.

**P** (Plan):

1. Given the concerning nature of the nipple discharge and previous lump, an urgent breast MRI is recommended for a comprehensive view.

2. Once MRI results are available, appropriate management or further diagnostics will be discussed.

3. Patient is encouraged to sign up on the patient portal for streamlined communication and result viewing.

4. Will review results with the patient as soon as they are available.

End of SOAP Note.

**Version 3:**

**CLINICAL NOTE**

**S**: *Subjective*

- Patient: Darby Washington

- Presenting Concerns: Patient reports nipple discharge that is "really, really dark brown, black" from her right breast for the past two weeks. She also notes a lump from the outside of her right breast that she noticed a few months ago, though an ultrasound at her annual checkup suggested it was probably benign. She reports some fatigue and an unintentional weight loss of a couple of pounds in the past two weeks.

- Past Medical History: Ms. Washington has never had any breast conditions previously. She has had a mammogram and an ultrasound, which did not show overly concerning findings.

- Medications: Occasional OTC Tylenol and Advil as needed.

- Social History: Smoked half a pack a day for 15-20 years, but quit 10 years ago. Drinks alcohol occasionally. No recreational drug use.

- Family History: Father died of a stroke in his early 80s, mother passed away in a housefire, one healthy brother. No known family history of breast cancer.

**O**: *Objective*

- Vital Signs: Temp: 97.6°F, HR: 101 bpm, BP: 138/92 mmHg, RR: 19/min, SpO2: 98% on room air, Weight: 135 lbs, Height: 5'6".

- Breast Exam: Right breast showed extra glandularity in the upper and outer quadrant without a discernible firm mass. Black discharge expressed from the nipple upon examination. Left breast appeared normal.

- Imaging: Recent mammogram and ultrasound showed only benign findings.

**A**: *Assessment*

- Abnormal nipple discharge from the right breast.

- Palpable lump in the right breast, previously identified as probably benign on ultrasound.

- Intentional weight loss and fatigue in the context of these findings warrants further investigation.

**P**: *Plan*

- Urgent breast MRI to obtain a more detailed view of the right breast. Targeting to get the MRI done within a week.

- Once results are received, will determine the next steps for management.

- Recommended the patient to sign up for the patient portal for easy access to test results and communication.

**Note**: Patient was informed of the findings and the planned course of action. She expressed concern about the timeline of the MRI but was reassured that the test would be done as urgently as possible.

*Dr. Jen, Breast Surgeon*

**Case 8: Heart Failure**

**Version 1:**

**SOAP Note**

**Subjective:**

*Chief Complaint:* Trouble breathing

*History of Present Illness (HPI):*

Francis White is a 6’1”, 220lb male who presents with dyspnea since 6 days ago. Initially, it was present during exertion such as going up stairs but became constant and worsened in the past couple of hours. He describes it as feeling like there's a weight on his chest. He also reports coughing in the last couple of hours and has had to prop himself up with pillows to sleep. Denies any chest pain but mentions a feeling of heaviness. Reports racing heartbeat and intermittent lightheadedness and dizziness over the past couple of hours.

*Past Medical History (PMH):* Hypertension, AFib, Type II Diabetes Mellitus.

*Medications:*

Furosemide 40mg QD (prescribed yesterday and not taken yet),

Pioglitazone 15mg QD,

Metoprolol 25mg BID,

Lisinopril 40mg QD,

Metformin 500mg BID,

Apixaban 5mg BID.

*Allergies:* None reported

*Social History (SH):* Retired custodian, lives with wife, previously a heavy smoker (2 packs/day for 20 years, quit ~15 years ago in 2006), occasional alcohol consumption (2-3 drinks socially), denies drug use.

*Family History (FH):* Father died in his 60s due to heart disease, Mother died during childbirth, No siblings.

*Review of Systems (ROS):* Notably for dyspnea, palpitations, and lightheadedness.

**Objective:**

*Vitals:*

Temp: Normal,

HR: 98bpm (irregular rhythm),

BP: 189/106,

RR: 31/min,

O2 Sat: 90%.

*Physical Examination:*

- Dyspneic and tachypneic but speaking in full sentences.

- Presence of pitting edema in legs.

- Elevated JVP.

- Respiratory sounds reveal expiratory rhonchi and bilateral basilar crepitations.

- Cardiac auscultation revealed an S3 gallop.

**Assessment:**

Acute exacerbation of heart failure, possibly decompensated due to non-compliance with medication, based on history, physical findings, and presentation.

**Plan:**

1. Admit for further workup and monitoring.

2. Obtain a chest X-ray to confirm pulmonary congestion.

3. Consider starting IV diuretics to help with fluid overload.

4. Monitor cardiac enzymes and EKG to rule out any ischemic events.

5. Consider echocardiogram to assess ejection fraction and any structural abnormalities.

6. Reinforce the importance of medication compliance and possibly review medications to see if any adjustments are needed.

7. Once stabilized, consider referring to cardiology for further management.

Note: This patient should be closely monitored, and further interventions should be considered based on the progression of his condition and any new findings.

**Version 2:**

**Clinical Note in SOAP format:**

**Subjective:**

- *Chief Complaint:* Difficulty in breathing, that has worsened over the last couple of hours. Describes it as having a "weight on chest", though not painful.

- *History of Present Illness:* Onset 6 days ago, initially with exertion, especially with activities like climbing stairs. Symptomatic relief with rest initially, but has now become more constant over the past couple of hours. No lying flat at night, needs to prop himself up with pillows. Has a cough that developed over the last few hours.

- *Medications:*

- Furosemide 40mg daily (started yesterday, not yet taken)

- Apixaban 5mg twice daily

- Metformin 500mg twice daily

- Lisinopril 40mg daily

- Metoprolol 25mg twice daily

- Pioglitazone 15mg daily

- *Past Medical History:* Hypertension, Atrial Fibrillation, Type 2 Diabetes.

- *Social History:* Lives with wife, retired (previously custodial staff at Lincoln High School). Exercises mainly by doing household chores. Quit smoking approximately 15 years ago, used to smoke two packs a day for about 20 years. Social drinker. No recreational drug use. Consumes caffeine in the morning.

- *Family History:* Father had heart disease and died in his 60s. Mother passed away during childbirth. No siblings.

**Objective:**

- *Vitals:*

- Temperature: Normal

- Heart Rate: 98 (irregular rhythm)

- Blood Pressure: 189/106

- Respiratory Rate: 31/min

- Oxygen Saturation: 90%

- Height: 6'1''

- Weight: 220lbs

- *Physical Exam:*

- Appears dyspneic and tachypneic but conversing in complete sentences.

- Appears anxious.

- Mild pitting edema in the legs.

- Elevated Jugular Venous Pulsation.

- Lung sounds: Expiratory rhonchi with basilar crepitations bilaterally.

- Heart sounds: Noted S3 gallop.

- Other exam normal.

**Assessment and Plan:**

- *Assessment:* Patient with a history of hypertension, AFib, and type 2 diabetes presenting with acute on chronic dyspnea, along with clinical signs suggestive of fluid overload. The clinical picture is consistent with acute heart failure exacerbation.

- *Plan:*

1. Begin diuretic therapy: Initiate Furosemide as prescribed.

2. Close monitoring of vitals and fluid status.

3. Recommend echocardiogram to assess cardiac function and structure.

4. Review and adjust hypertension and AFib medications as necessary.

5. Admit for close observation and further management.

6. Patient education about heart failure and its management.

**Note**: This clinical note is generated based on the information provided in the patient-doctor conversation. Additional diagnostic tests, treatments, and consultations may be necessary based on the patient's clinical status and the treating physician's clinical judgment.

**Version 3:**

**CLINICAL NOTE**

**Subjective**:

Mr. Francis White, a retired custodial staff member from Lincoln High School, presents with increasing dyspnea that began roughly six days ago and significantly worsened within the last few hours. He reports that initially he felt shortness of breath only with exertion such as ascending stairs. However, his breathing troubles have become persistent in the recent hours, making it difficult even at rest. He does not describe chest pain, but a sensation of weight or heaviness on the chest. He reports a cough that has developed in the last few hours. He denies having fever but confirms the nurse checked his temperature. When asked about orthopnea, he notes that he required multiple pillows to sleep the previous night. There is no similar past episode. Mr. White reports regular medications: furosemide 40mg daily, pioglitazone 15mg daily, metoprolol 25mg twice daily, lisinopril 40mg daily, metformin 500mg twice daily, and apixaban 5mg twice daily. His medical history includes hypertension, atrial fibrillation, and type 2 diabetes. He is up-to-date with his vaccinations including pneumonia, flu, and COVID. The patient denies any allergies. He lives with his wife and is currently retired. He used to smoke two packs a day but quit roughly 15 years ago. He drinks socially on weekends and consumes caffeine. Family history is notable for his father having heart disease and dying in his 60s.

**Objective**:

- Vitals: Temp - Normal, HR - 98 bpm (irregularly irregular), BP - 189/106 mmHg, RR - 31 breaths/min, O2 sat - 90%, Height - 6'1'', Weight - 220 lbs.

- General: In distress, tachypneic but conversing in full sentences, appears anxious.

- Cardiovascular: Irregularly irregular rhythm, S3 gallop noted.

- Respiratory: Expiratory rhonchi, bilateral basilar crepitations.

- Extremities: Mild pitting edema bilaterally.

- Neck: Elevated JVP.

**Assessment & Plan**:

The patient's presentation, physical examination, and history are consistent with an acute exacerbation of heart failure. The irregularly irregular rhythm noted on examination, combined with his history, indicates that his atrial fibrillation may be contributing. His medication list includes several agents for chronic medical conditions, including one (furosemide) that was newly prescribed and not yet started, indicating a recent concern about fluid retention.

**Plan**:

1. Start diuresis with IV furosemide for volume overload.

2. Obtain an EKG to evaluate rhythm and look for evidence of ischemia.

3. Check a set of labs, including BNP, CBC, BMP, liver function tests, and a TSH.

4. Consider an echocardiogram to assess cardiac function and structure.

5. Admit to the cardiology service for management of heart failure exacerbation and rate/rhythm control.

6. Patient education regarding medication compliance, fluid restriction, and monitoring for symptoms of worsening heart failure.

This note documents a thorough evaluation of a patient presenting with an acute exacerbation of heart failure. Immediate interventions should be initiated to stabilize the patient, with a more detailed plan to be formulated based on diagnostic results.

**Case 9: Polymyalgia Rheumatica**

**Version 1:**

**Clinical Note - SOAP Format**

**Subjective:**

*Nia Wallace presents with generalized soreness over the last four months, most pronounced in the shoulders, thighs, and calves. Also experiencing neck stiffness and pain. Describes the pain as achy and sore. Pain is worse in the morning and tends to improve as she becomes more active throughout the day. Reports difficulty in getting out of bed and standing up from a seated position without support. No associated fever, chills, night sweats, weight loss, rashes, headaches, vision changes, jaw pain, scalp tenderness, or joint swelling. Patient takes fluticasone and albuterol for asthma and supplements with calcium and Vitamin D. Medical history includes asthma, irritable bowel syndrome, breast cancer in remission, and high cholesterol. No statin due to muscle aches and cramps. Allergic to statin and penicillin (causes rash and throat swelling). Former smoker, occasional alcohol consumption, no current drug use. Family history is non-contributory with the exception of a father who passed away due to an accident.*

**Objective:**

*Vital signs:*

- Temperature: 97.6°F

- Heart rate: 91 bpm, regular rhythm

- Blood Pressure: 138/88 mmHg

- Respiratory rate: 19 breaths/minute

- O2 saturation: 98% on room air

- Weight: 105 pounds

- Height: 5 feet

*Physical examination:*

- Patient is moderately built, alert, oriented, in no acute distress but appears in some discomfort.

- No signs of anemia or scleral icterus.

- Head and Neck: No scalp or temple tenderness. No lymphadenopathy or tender temporal artery.

- Cardiovascular: Regular rate, normal S1 and S2, no murmurs.

- Respiratory: Good effort, normal sounds in all lung fields, no wheezes or crackles.

- Musculoskeletal: Difficulty standing up without support, tenderness in mentioned areas.

**Assessment:**

*Generalized musculoskeletal pain and stiffness, most prominent in the shoulders, thighs, and calves. Difficulty rising from a seated position. The patient has been experiencing these symptoms for about four months. The differential diagnosis would include polymyalgia rheumatica, fibromyalgia, myopathy due to medications, and other musculoskeletal disorders.*

**Plan:**

1. Recommend basic blood work, including Complete Blood Count (CBC), Erythrocyte Sedimentation Rate (ESR), and Creatine Phosphokinase (CPK) to evaluate for inflammatory conditions and myopathy.

2. Physical therapy referral for strengthening exercises and improving mobility.

3. Re-evaluate in 2-4 weeks to monitor progress and symptoms.

4. Advise patient to maintain an active lifestyle as tolerated, as it seems to alleviate some of the symptoms.

5. Discuss potential benefits of adding non-steroidal anti-inflammatory drugs (NSAIDs) or other pain-relieving medications if needed.

6. Consider consultation with rheumatology based on lab results and clinical course.

*Note: The plan is just an example based on the limited information given, and it's important to have a comprehensive assessment in a real-life scenario before creating a treatment plan.*

**Version 2:**

**Clinical Note**

*Subjective:*

**Chief Complaint**: Soreness and stiffness predominantly in shoulders, thighs, calves, and neck for the last 4 months.

**History of Present Illness (HPI)**:

- The pain is described as achy and sore.

- Worse in the morning but seems to improve with activity throughout the day.

- Difficulty arising from a sitting position or bed and often needs support.

- No associated fever, chills, night sweats, weight loss, rashes, headaches, vision changes, jaw pain while chewing, tenderness on scalp, or joint swelling.

**Past Medical History (PMH)**:

- Asthma (taking fluticasone and albuterol inhalers).

- Irritable bowel syndrome.

- Breast cancer (in remission).

- High cholesterol (no statin due to associated aches and cramps).

**Medications**:

- Fluticasone inhaler

- Albuterol inhaler

- Over-the-counter: Calcium and Vitamin D supplements.

**Allergies**:

- Statins (causes aches and cramps)

- Penicillin (causes rash and throat swelling).

**Social History (SH)**:

- Lives with husband.

- Non-smoker for 30 years.

- Alcohol consumption: 1-2 shots or beers 2-3 times a week.

- Past marijuana use in college.

- Occupation: Fashion designer at Cooper Union.

- Hobbies: Thrift shopping.

**Family History (FH)**:

- Father passed away at 40 due to a fall.

- Mother alive and well at 80.

- Sibling in Spain.

*Objective:*

**Vital Signs**:

- Temp: 97.6°F

- HR: 91 bpm

- BP: 138/88 mmHg

- RR: 19/min

- O2 Sat: 98% on room air

- Weight: 105 lbs

- Height: 5’

**Physical Examination**:

- Patient alert and oriented, appears in discomfort.

- HEENT: No anemia, no scleral icterus. No scalp tenderness or temple tenderness. No palpable lymphadenopathy.

- Cardiovascular: Regular rhythm, S1 and S2 normal, no murmurs.

- Pulmonary: Clear to auscultation bilaterally, no wheezes or crackles.

- Musculoskeletal: Difficulties getting up from a sitting position, needed support.

*Assessment & Plan:*

- **Muscle stiffness and soreness**: Given the symptoms are worse in the morning and improve with activity, and no other associated red flag symptoms, further workup is indicated to rule out conditions like polymyalgia rheumatica or other inflammatory conditions.

- **Hypertension**: Mildly elevated blood pressure on today’s visit. Recommend monitoring blood pressure and consider lifestyle modifications.

- **High Cholesterol**: Since the patient reported muscle aches with statins, alternative therapies or further monitoring might be considered.

Further diagnostic tests, lab works, or imaging may be ordered based on the initial findings. Referrals to specialists such as rheumatology or physiotherapy may also be considered based on the further assessments.

*Note*: This is a SOAP note based on the provided conversation. Any treatment plans or further actions should be tailored to the patient's specific situation and based on thorough clinical examination and judgment.

**Version 3:**

**SOAP Note**

**Subjective:**

- Chief Complaint: Generalized aches and soreness, particularly in the shoulders, thighs, calves, and neck stiffness.

- Duration: Symptoms present for approximately 4 months.

- Quality: Described as "achy" and "sore".

- Timing: Worse in the morning but improves with activity throughout the day.

- Aggravating factors: Standing up from a seated position or bed requires support.

- Medications:

- Prescription: Fluticasone and Albuterol inhalers for asthma.

- OTC/Supplements: Calcium and Vitamin D.

- Past Medical History:

- Asthma

- Irritable bowel syndrome

- Breast cancer in remission

- High cholesterol. Statin not tolerated due to muscle aches and cramps.

- Allergies: Statins and penicillin. Penicillin causes rash and throat swelling.

- Social History:

- Lives with husband.

- Non-smoker for >30 years.

- Drinks 1-2 shots or beers a few times a week.

- Occupation: Fashion designer at Cooper Union.

- Hobbies: Thrift shopping.

- Family History:

- Father passed away in an accident.

- Mother alive and well at 80 years.

- One sibling in Spain.

**Objective:**

- Vitals:

- Temperature: 97.6°F

- Heart rate: 91 bpm, regular rhythm

- Blood pressure: 138/88 mmHg

- Respiratory rate: 19 breaths/minute

- Oxygen saturation: 98% on room air

- Weight: 105 lbs

- Height: 5’0”

- General appearance: Moderately built, alert, oriented, appears in some discomfort but not in acute distress.

- HEENT: No tenderness of the scalp, temples or neck. No palpable lymphadenopathy. No scleral icterus.

- Cardiovascular: Normal S1, S2, regular rate, no murmurs.

- Respiratory: Good respiratory effort with normal breath sounds. No wheezes or crackles.

- Musculoskeletal: Difficulty standing up from a seated position without support.

**Assessment:**

- Generalized musculoskeletal pain and stiffness of unclear etiology. The nature of the symptoms, particularly the morning stiffness which improves with activity, is suggestive of a possible inflammatory condition. Differential diagnoses include rheumatoid arthritis, polymyalgia rheumatica, and other connective tissue diseases.

**Plan:**

1. Order labs including:

- CBC

- ESR

- CRP

- Rheumatoid Factor

- Anti-CCP

2. Recommend over-the-counter NSAIDs for pain relief and stiffness.

3. Schedule a follow-up in 2 weeks to discuss the results of the tests and potential treatment plans.

4. Educate the patient on the importance of physical activity and staying active.

5. Encourage the patient to monitor symptoms and report any worsening or new symptoms immediately.

Note: Further sections of the physical examination and additional recommendations may be provided after completing the examination and assessing the results.

**Case 10: Congestive Heart Failure**

**Version 1:**

**CLINICAL NOTE: SOAP Format**

**Subjective:**

- Chief Complaint: Pat Tynes, a patient presenting with shortness of breath on exertion for the past couple of months.

- HPI (History of Present Illness): The patient reports experiencing fatigue and leg swelling just above the ankle. No history of fever, cough, sore throat, or exposure to sick individuals. Denies experiencing palpitations or dizziness. Finds difficulty breathing when lying flat, hence uses two pillows to sleep.

- Past Medical History: Diabetes type II, high blood pressure, and atrial fibrillation.

- Medications: Metoprolol (for BP and irregular heartbeat), Lisinopril (for high BP), Metformin & Pioglitazone (for diabetes), Apixaban (for AFib).

- Past Surgical History: None.

- Social History: Smoked 2 packs/day for approximately 20 years, quit 15 years ago. Consumes 2-3 glasses of wine weekly. No recreational drug use.

- Family History: Mother died during childbirth. Father passed away from a heart attack at age 60. No siblings.

**Objective:**

- Vital Signs: Temp: 97.3°F, HR: 68 bpm, BP: 149/96 mmHg, RR: 25/min, SpO2: 96% on room air, Height: 5’, Weight: 105 lbs.

- Physical Exam:

* Heart: Regular rhythm.

* Lungs: Abnormal sounds noted.

* Neck: No mention.

* Extremities: Edema noted in ankles.

**Assessment:**

- Patient may have congestive heart failure based on the clinical presentation of leg swelling, shortness of breath on exertion, and abnormal lung sounds. Additional history, including the previous episode of atrial fibrillation, hypertension, and diabetes, places the patient at higher risk for cardiac issues.

**Plan:**

- Laboratory: Comprehensive blood work.

- Imaging: Chest x-ray.

- Cardiac: EKG and echocardiogram to assess cardiac structure and function.

- Follow-Up: Pending results, may need cardiology referral and potential medication adjustments.

**Discussion:**

The patient was informed about the possible diagnosis of congestive heart failure and its implications. The plan for further tests was explained, and the patient was reassured. Immediate interventions and follow-ups will be based on the results of the investigations.

*End of Note*

**Version 2:**

**Clinical Note (SOAP Format)**

**Subjective (S):**

*Chief Complaint*: Shortness of breath, unusual fatigue, and leg swelling for a few months.

*Past Medical History*: Diabetes type 2, hypertension, atrial fibrillation (AFib).

*Medications*:

- Metoprolol (for hypertension and arrhythmia)

- Lisinopril (for hypertension)

- Metformin (for diabetes)

- Pioglitazone (for diabetes)

- Apixaban (for AFib)

*Social History*: Smoked two packs a day for about 20 years but quit 15 years ago. Drinks 2-3 glasses of wine per week. No recreational drugs. No over-the-counter medications.

*Family History*: Mother died during childbirth. Father had a heart attack at age 60. No siblings.

*Review of Systems (ROS)*:

- Shortness of breath on exertion (e.g., climbing stairs)

- Requires an extra pillow to sleep (potential orthopnea)

- No fevers, chills, cough, sore throat, or nasal congestion.

- No palpitations, dizziness, or chest pain.

**Objective (O):**

*Vitals*: Temp - 97.3°F, HR - 68 bpm, BP - 149/96 mmHg, RR - 25 breaths/min, O2 Sat - 96% on room air, Height - 5', Weight - 105 pounds.

*Physical Examination*:

- Elevated neck veins.

- Lung sounds: Abnormal sounds suggesting possible fluid.

- Cardiac sounds: No noted murmurs or irregularities during this encounter.

- Abdomen: Normal bowel sounds.

- Extremities: Bilateral ankle edema noted.

**Assessment and Plan (A&P):**

*Assessment*:

- Suspected congestive heart failure (CHF) based on presenting symptoms, physical examination findings of bilateral ankle edema, elevated neck veins, and abnormal lung sounds. History of AFib and hypertension is notable as these are risk factors.

*Plan*:

- Order lab tests including a basic metabolic panel (BMP), complete blood count (CBC), B-type natriuretic peptide (BNP) to assess for heart failure.

- Chest X-ray to evaluate for pulmonary congestion.

- Echocardiogram to assess heart structure and function.

- EKG to assess cardiac rhythm.

- Discuss potential changes in medication or introduction of diuretics based on results.

- Follow-up appointment in 1-2 weeks or sooner based on results.

- Counsel on importance of daily weight monitoring and salt restriction.

**Note**: It's crucial that the patient be advised of the seriousness of these findings and understand the next steps. Education on CHF, its causes, symptoms, and management will be important. It's also crucial to ensure close follow-up to address the test results and implement the appropriate treatment plan.

**Version 3:**

**SOAP NOTE:**

**S**ubjective:

- Chief Complaint: Shortness of breath on exertion, unusual tiredness or fatigue, and leg swelling just above the ankle. These symptoms have been present for a couple of months and have been progressively getting worse.

- Past Medical History: Diabetes type two, high blood pressure, and atrial fibrillation (AFib).

- Medications: Metoprolol (for high blood pressure and irregular heartbeat), Lisinopril (for high blood pressure), Metformin and Pioglitazone (for diabetes), and Apixaban (for AFib).

- Social History: Former smoker (two packs/day for 20 years, quit 15 years ago). Drinks 2-3 glasses of wine per week. No recreational drug use.

- Family History: Mother died at childbirth, father died of a heart attack at age 60. No siblings.

**O**bjective:

- Vital Signs: Temp: 97.3°F, HR: 68 bpm, BP: 149/96 mmHg, RR: 25 breaths/min, SpO2: 96% on room air.

- Height: 5'0", Weight: 105 lbs.

- Physical Exam: Elevated neck veins, abnormal lung sounds suggestive of fluid, ankle edema present, and heart sounds normal.

**A**ssessment:

Possible congestive heart failure (CHF) based on patient symptoms (shortness of breath, fatigue, leg swelling) and physical findings (elevated neck veins, abnormal lung sounds, and ankle edema).

**P**lan:

1. Recommend bloodwork to check for cardiac markers and other relevant tests.

2. Chest X-ray to evaluate for any pulmonary congestion or other abnormalities.

3. Echocardiogram to evaluate heart function, especially given the patient's history of AFib.

4. Electrocardiogram (EKG) to assess the heart's electrical activity and to check for any abnormalities or changes.

5. Discuss potential treatments and management strategies once results from the above tests are available.

Note: It's essential to monitor the patient closely and provide education on what congestive heart failure is, potential causes, and the importance of follow-up.

**Case 11: Decreased Fetal Movement**

**Version 1:**

**Clinical SOAP Note**

**Subjective:**

*Rhea Michaels, a 32-year-old G1P0 at 35 weeks gestation, presents with a chief complaint of decreased fetal movements since midnight. She is a known case of Type 2 Diabetes Mellitus and is on insulin, taken twice daily. Patient states she has had no other pregnancies, miscarriages, or abortions. She denies any contractions, rupture of membranes, vaginal bleeding, headaches, visual changes, or abdominal pain. The patient reported a normal last menstrual period approximately 8 months ago and her menstrual cycles are typically every 20-30 days lasting for 3-4 days. Her last Pap smear in 2020 was initially abnormal but follow-up was normal. She is allergic to aspirin and reports having hay fever. Rhea lives with her partner and has a pet dog. She is currently working from home as an accountant. She denies tobacco, alcohol, and recent marijuana use, though she used marijuana a few weeks ago due to nausea. Her prenatal medication list includes insulin, Zofran, and prenatal vitamins. She has no known surgical history.*

**Objective:**

- **Vitals**: BP 138/84, Pulse 82, Oxygen saturation 110% on room air

- **Weight**: Current weight is 155lbs, pre-pregnancy weight was 110lbs

- **Physical Examination**:

- Heart: Regular rate and rhythm, no murmurs

- Lungs: Clear bilaterally

- Abdomen: Gravid, soft, nontender, nondistended

- Fundal Height: Measuring 32 weeks, 3 weeks behind gestational age

- Lower Extremities: Warm, well perfused, no peripheral edema

- **Ultrasound**: Single live intrauterine pregnancy in cephalic presentation, adequate amniotic fluid (9cm), anterior placenta. Biophysical profile is 8/8.

**Assessment/Analysis**:

- Decreased fetal movements

- Pregnancy with Type 2 Diabetes

- Fundal height smaller than gestational age

- No reactive non-stress test but a reassuring Biophysical Profile

- Possible urinary tract infection (as per dipstick results)

**Plan**:

1. Continuous fetal monitoring overnight for fetal wellbeing.

2. Formal ultrasound in the morning to rule out intrauterine growth restriction given the discrepancy between the fundal height and gestational age.

3. Follow up on urine cultures to confirm/rule out UTI.

4. Counsel patient on the importance of blood sugar control and managing diabetes during pregnancy. Provide additional information and resources.

5. Reassure patient and provide guidelines on monitoring fetal movements and when to seek medical attention.

6. Consider further follow-ups or interventions based on overnight monitoring and ultrasound findings.

*Note: A SOAP note format typically consists of a Subjective section (what the patient says), Objective section (what the healthcare provider observes and measures), Assessment or Analysis section (the diagnosis or problem list), and the Plan (what needs to be done next).*

**Version 2:**

Clinical SOAP Note:

**Subjective:**

- **Chief Complaint (CC):** Decreased fetal movement since midnight.

- **History of Present Illness (HPI):** Ms. Rhea Michaels, a 32-year-old G1P0 at 35 weeks gestation, noticed decreased fetal movement around midnight. Upon noticing, she reached out to the OB nurse hotline who advised her to come in for an evaluation.

- **Past Medical History (PMH):** Type 2 Diabetes Mellitus, treated with insulin twice daily. Has a history of hay fever. Reported an abnormal Pap smear in 2020 but a subsequent one was normal.

- **Medications:** Insulin, Zofran, and a prenatal vitamin.

- **Allergies:** Aspirin, Hay fever.

- **Family History:** Father with hypertension, mother with diabetes, and an aunt with kidney cancer.

- **Social History:** Lives with her partner and a dog. She is currently employed as an accountant, working from home. Denies alcohol and tobacco use. Recently stopped marijuana use due to nausea. Denies any safety concerns at home.

**Objective:**

- **Vitals:** BP: 138/84, Pulse: 82, Weight: 155 pounds (pre-pregnancy weight: 110 pounds), Oxygen saturation: 110% on room air.

- **General:** No acute distress.

- **Cardiovascular:** Regular rate and rhythm, no murmurs, rubs, or gallops.

- **Pulmonary:** Clear to auscultation bilaterally.

- **Abdominal:** Soft, non-tender, gravid with a fundal height measuring 32 cm.

- **Extremities:** Warm, well-perfused, no peripheral edema.

- **Fetal Monitoring:** Baseline fetal heart rate of 130, moderate variability, no accelerations, no decelerations.

- **Ultrasound:** Single cephalic fetus, adequate amniotic fluid (9cm), anterior placenta. Biophysical profile score: 8 out of 8.

**Assessment:**

- 35-week pregnant female with concerns of decreased fetal movement.

- Measurements on physical exam slightly smaller than gestational age (32 cm at 35 weeks).

- Nonreactive non-stress test but reassuring biophysical profile.

- Type 2 Diabetes Mellitus.

**Plan:**

1. Monitor fetus for at least 2 more hours.

2. Order a formal growth ultrasound for the morning to assess if the fetus is growth restricted.

3. Monitor and await results of urine cultures due to the presence of pyuria, to rule out a UTI.

**Note:** SOAP (Subjective, Objective, Assessment, Plan) is a common method of documenting patient encounters in healthcare settings. This format ensures that the clinician captures the essential details of the patient's presentation, clinical findings, differential diagnosis, and management in a structured manner.

**Version 3:**

**Clinical Note (SOAP Format)**

**Subjective:**

32-year-old G1P0 woman, Rhea Michaels, at 35 weeks gestation presents to the office reporting decreased fetal movement since midnight. She denies any vaginal bleeding, contractions, rupture of membranes, headaches, vision changes, or intense right-sided pain. Patient has a history of Type 2 Diabetes Mellitus and is on insulin twice a day. She also reports having hay fever and is allergic to aspirin. She denies any history of miscarriages or abortions. She uses Zofran for nausea and has stopped using marijuana a few weeks ago, which she had used for nausea as well. Patient denies any history of smoking, tobacco use, or alcohol consumption. She lives with her partner and a dog. The patient denies any past medical or surgical history, aside from the above mentioned. No known history of domestic violence.

**Objective:**

- Vital Signs: BP 138/84, Pulse 82, Oxygen Saturation 110%

- Weight: Pre-pregnancy 110 lbs, Current 155 lbs

- Physical Examination: Appears anxious but in no acute distress, clear lung and heart sounds, soft and non-tender abdomen, gravid uterus measures 32 cm fundal height.

- Fetal Heart Tracing: Baseline 130 with moderate variability, no accelerations, or decelerations.

- Ultrasound: Single fetus in cephalic presentation, adequate amniotic fluid at 9cm, anterior placenta. Biophysical profile (BPP) score of 8/8.

- Urinalysis: Noted pyuria.

**Assessment:**

- 35 weeks pregnant with decreased fetal movement.

- Type 2 Diabetes Mellitus.

- Potential intrauterine growth restriction given discrepancy between gestational age and fundal height.

- Possible urinary tract infection (UTI) based on urinalysis findings.

**Plan:**

1. Monitor fetal wellbeing overnight.

2. Continue monitoring for 2 hours for fetal movements.

3. Non-stress test was non-reactive, hence proceeding with BPP, which was reassuring.

4. Schedule a formal ultrasound for the morning to assess for intrauterine growth restriction.

5. Follow-up on urine cultures to assess for UTI.

6. Counsel patient regarding findings and plan for care.

*Note: A complete plan and further discussions with the patient should be documented. Given the concerns of decreased fetal movement, close monitoring and further assessments as necessary should be conducted.*

**Case 12: Diverticulitis**

**Version 1:**

**SOAP Note**

**Subjective:**

- **Chief Complaint (CC):** Abdominal pain for about one week.

- **History of Present Illness (HPI):** Ms. Joan Hoffart is a retired schoolteacher who reports that the abdominal pain started about a week ago, gradually increasing in intensity over time. The pain is described as a dull ache, mostly located in the left side. It occasionally becomes very painful. Over the past week, she noticed darker (mahogany) bowel movements and has experienced some nausea. Ms. Hoffart reports being more easily able to have bowel movements lately and experiences pain before defecating. She took her temperature this morning and it was 100.2°F.

- **Past Medical History (PMH):** Diabetes type 2 (on metformin 1000mg BID), Hypothyroidism (on levothyroxine), previous history of a similar pain a couple of years ago (hospitalized and treated for bowel infection with IV antibiotics), gallbladder removal surgery 10 years ago.

- **Medications:** Metformin 1000mg BID, Levothyroxine.

- **Allergies:** Bactrim.

- **Social History (SH):** Non-smoker, occasional alcohol (sips during religious events), no recreational drug use. Recently retired schoolteacher starting a part-time job soon.

- **Family History (FH):** Mother has Diabetes type 2. Father had prostate cancer and COPD.

- **Review of Systems (ROS):** No vomiting, but nausea since yesterday. She has felt chills and fluctuations in temperature since this morning. Reduced appetite. Bowel movements have become slightly looser than her usual constipation, and they are darker in color.

**Objective:**

- **Vital Signs:** Reported fever of 100.2°F at home.

- **Physical Examination:** (Anticipated) Abdominal exam will focus on locating the point of maximum tenderness, signs of inflammation, and any masses. Also, evaluation of bowel sounds, guarding, and rigidity will be noted.

**Assessment:**

- **Primary Diagnosis:** Abdominal pain, left lower quadrant. Differentials include diverticulitis (especially given her history), IBS, colitis, or other gastrointestinal disorders.

- **Secondary Diagnosis:** To be determined based on further workup and tests.

**Plan:**

1. **Diagnostic Tests:** Recommended abdominal CT scan or ultrasound to visualize the abdomen and possible areas of inflammation or infection.

2. **Treatment:** If diverticulitis is confirmed, a course of antibiotics might be started based on her past experience.

3. **Follow-up:** Schedule a follow-up appointment in one week or sooner if symptoms worsen.

4. **Additional Recommendations:** Based on her concerns about her husband, consider referring them to a neurologist or geriatric specialist for evaluation of possible cognitive issues.

*Please note that this SOAP note is a simulation based on the presented scenario and might differ from an actual clinical note.*

**Version 2:**

**Clinical Note (SOAP Format)**

**Subjective:**

**Patient:** Joan Hoffart

- **Chief Complaint:** Acute left-sided abdominal pain for about a week that has progressively worsened.

- **History of Present Illness:** Patient noticed pain about a week ago, which was initially mild but has since been progressively worsening. The pain is described as a dull ache but becomes sharp and severe at times. Pain localized to the left side of the abdomen, does not radiate elsewhere. Accompanied by decreased appetite, nausea (no vomiting), a change in bowel habits (previously constipated but recently looser stools), and pain before bowel movements which relieves after. Noticed stool color to be mahogany (a change from usual). Additionally, experienced fever and chills this morning with a recorded temperature of 100.2°F.

- **Past Medical History:** Diabetes Mellitus Type 2 (managed with Metformin 1000mg twice daily), Hypothyroidism (on Levothyroxine). Has history of similar pain that led to hospitalization a couple of years ago; diagnosed with an infection in the bowels and received IV antibiotics. No recent or recurrent episodes until now.

- **Surgical History:** Cholecystectomy (gallbladder removed) via laparoscopy approximately ten years ago.

- **Medications:** Metformin 1000mg twice daily for diabetes, Levothyroxine for hypothyroidism.

- **Allergies:** Bactrim (resulted in an itchy rash in childhood).

- **Family History:** Mother with Diabetes Mellitus Type 2, Father with Prostate Cancer and COPD.

- **Social History:** Retired schoolteacher. Does not consume alcohol regularly, non-smoker, denies drug use. Husband showing signs of forgetfulness and recently had instances of confusion.

**Objective:**

**Physical Examination:** (Note: Examination details not provided in the conversation. Here is a placeholder based on the given history.)

- **General:** Alert, oriented, in no acute distress.

- **Abdomen:** Tender to palpation in the left lower quadrant. No guarding or rigidity. No palpable masses. Normal bowel sounds.

**Assessment and Plan:**

- **Differential Diagnosis:** Diverticulitis, Irritable Bowel Syndrome (IBS), Inflammatory Bowel Disease (IBD), Gastrointestinal infection.

- **Plan:** Recommend labs (CBC, BMP, CRP) and abdominal imaging to further evaluate. Initiate treatment based on results and clinical evaluation. Advise the patient on dietary precautions and to follow up for any worsening of symptoms or new concerns.

**Note:** Further assessment and clinical findings from the physical examination will be essential to narrow down the diagnosis and guide management.

**Version 3:**

**Clinical Note: SOAP Format**

**S**ubjective:

- **Chief Complaint**: Abdominal pain, worsening over a week, with intermittent severe episodes.

- **HPI**: The patient, Joan Hoffart, describes the pain as a "dull ache" localized to the left side of her abdomen. She reported feeling nauseous since yesterday, decreased appetite, and some changes in bowel movements. Normally constipated, she reported her stools are somewhat looser and have been darker (mahogany) in color than usual. She also noted some discomfort prior to bowel movements but relief after. This morning she woke up with a temperature of 100.2°F, chills, and alternating hot and cold sensations.

- **Past Medical History**: Diabetes type 2 (on metformin 1000mg twice daily), Hypothyroidism (on levothyroxine), Diverticulosis diagnosed via colonoscopy.

- **Past Surgical History**: Cholecystectomy via laparoscopic surgery approx. 10 years ago, a previous hospitalization for an infection in the bowels treated with IV antibiotics.

- **Medications**: Metformin, Levothyroxine.

- **Allergies**: Bactrim (caused rash).

- **Family History**: Mother with diabetes type 2; father with prostate cancer and COPD.

- **Social History**: Retired schoolteacher starting part-time clerical work next week. Lives with husband, Bill, who shows signs of forgetfulness. Three children, all grown and living away. Has a dog, Pipi-bark-bark. Minimal alcohol consumption and no history of smoking or recreational drug use.

- **Additional Information**: Concerns about husband's recent forgetfulness. Recalls a past episode similar to the current symptoms a couple of years ago.

**O**bjective:

*Vital Signs and Measurements*: [Need physical examination details]

*Physical Exam*: [Pending]

**A**ssessment:

1. Acute abdominal pain, left side, worsening over one week.

2. Possible diverticulitis or another recurrence of previous bowel infection, considering the past history and the presentation.

3. Febrile episode, possibly related to the abdominal pain.

4. Concern for husband's cognitive decline.

**P**lan:

1. Recommend abdominal imaging (CT or Ultrasound) to visualize any inflammation or abnormalities.

2. Blood work, including CBC, CRP, and other inflammatory markers.

3. Consider antibiotics based on the result of imaging and lab tests.

4. Monitor temperature and pain level.

5. Consider a referral for the husband's cognitive evaluation or geriatric consult.

6. Follow up after results are in or if symptoms worsen.

---

**Note**: Above SOAP note is based on the provided conversation and might require further updates after the physical examination and any subsequent diagnostics.

**Case 13: Scleroderma**

**Version 1:**

Clinical Note in SOAP format:

**S: (Subjective)**

- Chief Complaint: Preoperative Evaluation for upcoming gallbladder surgery due to gallstones.

- History of Present Illness: Veronica Jackson, a wind turbine worker, presents with upper right abdominal pain for 2 weeks. Imaging shows gallstones. Patient denies nausea, vomiting, fevers, or chills. Reports a recent change in gait, feeling like she's walking slower, but denies shortness of breath or chest pain.

- Past Medical History: Denies heart problems, stroke, diabetes, kidney disease, and liver disease.

- Medications: Over-the-counter multivitamin, occasional Tylenol or aspirin.

- Social History: Non-smoker, non-drinker, no recreational drug use. Lives with wife.

- Family History: Father with stable angina. Grandmother (paternal side) had Parkinson's disease. No known family history of anesthesia reactions.

**O: (Objective)**

- Vital Signs: Temp: 36.8°C, HR: 89, BP: 134/82, RR: 17, Weight: 189 lbs, Height: 5'8''.

- Physical Examination:

* General: Alert, oriented, no acute distress, no jaundice.

* Cardiovascular: Normal heart sounds, no murmurs.

* Respiratory: Normal bilateral air entry, no abnormal sounds.

* Abdominal: Soft, mildly tender in the right upper quadrant, no rebound or guarding.

* Musculoskeletal: Thickened and hardened skin over the back of hands and fingers.

* Neurological: Normal mental status, cranial nerves, and strength. Full range of motion in upper extremities and slightly restricted knee flexion. No tremors.

**A: (Assessment)**

1. Gallstones with associated upper right abdominal pain. Gallbladder surgery scheduled in 14 days.

2. Altered gait - etiology unclear but Parkinson's seems unlikely.

3. Abnormal skin findings suggestive of scleroderma or systematic sclerosis.

**P: (Plan)**

1. Proceed with gallbladder surgery as scheduled.

2. Additional testing for suspected scleroderma.

3. Patient education about potential autoimmune condition.

4. Reassurance regarding lack of tremors and reduced likelihood of Parkinson’s.

(Note: The plan will be expanded based on the testing results and the patient’s progress. Also, collaborative care with specialists like rheumatologists may be considered based on the results.)

**Version 2:**

**SOAP NOTE**

**Subjective:**

*Chief Complaint:* Preoperative evaluation for gallbladder surgery

*History of Present Illness:* Veronica Jackson is a patient scheduled for gallbladder surgery in 14 days due to the presence of gallstones. She reports upper right abdominal pain for two weeks, with no associated nausea, vomiting, fever, or chills. Imaging has confirmed the presence of gallstones.

*Past Medical History:* No known history of heart problems, stroke, diabetes, kidney disease, or liver disease. Has never had surgery or anesthesia. Currently, she takes an over-the-counter multivitamin and occasional Tylenol for the abdominal pain. She has noticed a recent change in her movement pattern, describing walking as slightly more difficult than before, though she couldn't specify the exact nature of the change.

*Family Medical History:* Father diagnosed with stable angina around age 70. Maternal grandmother had Parkinson’s disease.

*Social History:* Non-smoker. No alcohol or recreational drug use. Lives with wife.

*Review of Systems:* General - No weight loss. Cardiovascular - No chest pain, palpitations. Respiratory - No cough, dyspnea, or wheezing. Gastrointestinal - Right upper quadrant pain without associated gastrointestinal symptoms. Neurological - No tremors or weakness. Musculoskeletal - No joint pain but noticed a change in gait.

**Objective:**

*Vital Signs:*

- Temperature: 36.8°C

- Heart Rate: 89 bpm

- Blood Pressure: 134/82 mmHg

- Respiratory Rate: 17/min

- Weight: 189 lbs

- Height: 5'8''

*Physical Examination:*

- General: Alert and oriented x3, no acute distress

- Cardiovascular: Regular rhythm, no murmurs

- Respiratory: Clear to auscultation bilaterally

- Abdominal: Soft, mildly tender in right upper quadrant, no rebound or guarding

- Skin: Thickening and hardening over the back of hands and fingers

- Neurological: Normal mental status, cranial nerves intact, normal muscle strength and tone, slightly restricted flexion of knees, and no tremors

- Musculoskeletal: Noticed change in gait

**Assessment and Plan:**

1. **Cholelithiasis** - Plan to proceed with scheduled gallbladder surgery. Recommend clear communication with surgical team regarding anesthesia.

2. **Abnormal Gait & Skin Changes** - Suspect an autoimmune condition, possibly scleroderma or systematic sclerosis.

- Plan: Further testing to determine diagnosis. Consider referring to a rheumatologist for additional evaluation.

3. **Preoperative Evaluation** -

- Review lab results.

- Ensure anesthesia team is aware of her medical history.

- Counsel about pre and post-operative care, ensuring her wife is available for support.

**Plan:** Proceed with the scheduled surgery while keeping an eye on her possible autoimmune condition. Ensure she understands the risks and benefits of the procedure and advise on the importance of post-operative care.

**Other Notes:** Given the family history of Parkinson’s and her concerns, though her symptoms don't clearly indicate Parkinson's, it would be wise to continue monitoring her neurological status and consider a neurology consult if symptoms progress.

**Version 3:**

**Clinical Note: Preoperative Evaluation in SOAP Format**

**S (Subjective)**:

Chief Complaint: Preoperative evaluation for upcoming gallbladder surgery.

History of Present Illness:

Ms. Veronica Jackson, a 5’8’’, 189-pound female, presents today for preoperative evaluation regarding a planned gallbladder surgery scheduled in 14 days due to gallstones. She has been experiencing right upper quadrant abdominal pain for the past two weeks without any associated nausea, vomiting, fevers, or chills.

Past Medical History: No known heart problems, stroke, diabetes, kidney or liver disease.

Medications: Over-the-counter multivitamin and occasional Tylenol or aspirin.

Social History:

Occupation: Wind turbine technician.

Alcohol/Drugs: Denies use.

Smoking: Non-smoker.

Living Situation: Lives with wife.

Family History: Father with stable angina, paternal grandmother had Parkinson's disease.

**O (Objective)**:

Vital Signs: Temp: 36.8°C, HR: 89 bpm, BP: 134/82 mmHg, RR: 17/min.

General Exam: Alert, oriented, no jaundice, and not in acute distress.

Cardiovascular: Normal heart sounds, no murmurs.

Respiratory: Bilateral equal air entry, no abnormal lung sounds.

Abdominal: Soft, mild tenderness in right upper quadrant without rebound or guarding.

Dermatological: Thickening and hardening of skin over the back of hands and fingers.

Neurological: Normal mental status and cranial nerves. Normal strength, muscle tone, and no tremor. Slightly restricted knee flexion bilaterally. Noticed slow and deliberate gait.

**A (Assessment)**:

1. Gallstones – Scheduled for surgery in 14 days.

2. Recent changes in gait and skin findings suggestive of an autoimmune condition - potential scleroderma/systematic sclerosis.

**P (Plan)**:

1. Proceed with gallbladder surgery as planned.

2. Workup for potential scleroderma: Recommend a full rheumatological evaluation and appropriate lab tests.

3. Recommend closer follow-up after the surgery to monitor and manage potential autoimmune condition.

4. Discuss potential implications and management strategies with the patient post-operatively.

**End of Note**.

**Case 14: Colon Cancer**

**Version 1:**

**SOAP Note for Ash Park**

**Subjective:**

- Chief Complaint: Shortness of breath for the past month and ongoing fatigue.

- Past Medical History: Anemia diagnosed six weeks ago (currently on iron supplements), high blood pressure (on lisinopril and amlodipine), high cholesterol (on rosuvastatin), type 2 diabetes (on metformin and pioglitazone), hypothyroidism (on thyroxine).

- Medications: Lisinopril, Amlodipine, Rosuvastatin, Metformin, Pioglitazone, Thyroxine, Iron supplements, Magnesium supplement.

- Allergies: None known.

- Social History: Recently moved from Boston to live with daughter; retired English teacher; doesn't smoke, drink, or use recreational drugs; enjoys painting.

- Family History: Father died from heart attack in his 50s (smoker with high blood pressure); Mother had type 2 diabetes and passed away in a car accident; Older brother died of colon cancer.

**Objective:**

- Vitals: Temp 96.4°F, HR 170 bpm, BP 118/82, RR 25 bpm, O2 sat 96%, Weight 220 pounds, Height 5’1’’.

- Physical Examination:

* General Appearance: Alert, oriented, appears comfortable.

* Cardiovascular: Regular rhythm, no murmurs.

* Respiratory: Normal breath sounds, no wheezes or crackles.

* Gastrointestinal: Abdomen soft, non-tender, no guarding, no ascites, no palpable masses.

* Musculoskeletal: Normal gait, though with short steps and slightly curved posture.

* Neurological: Alert, oriented x3, no reported dizziness or syncope.

**Assessment:**

- Primary concern of shortness of breath.

- Fatigue and anemia.

- Constipation with dark stools.

- Weight loss (approx. 10 lbs).

- No abdominal pain, bleeding, fever, coughing, chest pain, or dizziness.

- Concern for potential colon cancer due to family history.

**Plan:**

- Blood tests: CBC, Serum electrolytes with calcium and magnesium, Kidney function tests, Liver function tests, Thyroid function test (TSH), Lipid panel, BNP.

- Imaging: Chest x-ray, EKG, and Echocardiogram.

- Urgent GI referral for colonoscopy.

- Follow up: Await blood test results, review imaging results, and schedule colonoscopy. If symptoms worsen, advise patient to seek immediate medical attention.

Note: This SOAP note is based on the provided conversation. The medical assessments and plan should be considered in the context of this hypothetical scenario and not used for actual clinical decisions.

**Version 2:**

**Clinical SOAP Note**

**Subjective:**

*Chief Complaint:*

Ash Park, a patient, presents with a chief complaint of shortness of breath that has been ongoing for a month. The patient has also been experiencing fatigue and tiredness for a couple of months.

*History of Present Illness:*

- Shortness of breath for one month.

- Fatigue and tiredness for a couple of months.

- Constipation that is on and off.

- Appetite is reduced due to constipation leading to discomfort after eating.

- Weight loss – Patient has tightened belts and estimates a weight loss of about 10 pounds.

- Dark-colored stools, potentially due to iron supplements.

- No presence of blood in stools, abdominal pain, bleeding from elsewhere, fever, chills, coughing, chest pain, swelling in legs, dizziness, or palpitations.

*Medication:*

- Lisinopril and amlodipine for high blood pressure.

- Rosuvastatin for high cholesterol.

- Metformin and pioglitazone for type 2 diabetes.

- Thyroxine for hypothyroidism.

- Iron supplements for anemia.

- Magnesium supplements for leg cramps.

*Medical History:*

- High blood pressure

- High cholesterol

- Type 2 diabetes

- Hypothyroidism

- Anemia

**Objective:**

*Vitals:*

- Temperature: 96.4°F

- Heart rate: 170 beats per minute

- Blood pressure: 118/82 mm Hg

- Respiratory rate: 25 breaths/min

- Oxygen saturation: 96%

- Weight: 220 pounds

- Height: 5’1’’

*Physical Exam:*

- General: Alert and oriented. Warm, dry skin. No signs of distress.

- Cardiac: Normal S1, S2, no murmurs.

- Respiratory: Clear breath sounds, no wheezing.

- Abdominal: Soft, non-tender, no masses or ascites.

- Extremities: No edema.

**Assessment:**

- Shortness of breath and fatigue with a history of anemia. Given the family history of colon cancer in a sibling, dark stools, and recent weight loss, a gastrointestinal etiology including possible colon cancer is a concern.

**Plan:**

*Tests:*

- CBC

- Serum electrolytes with calcium and magnesium.

- Kidney function tests.

- Liver function tests.

- TSH for thyroid function.

- Lipid panel.

- BNP.

- Chest x-ray.

- EKG.

- Echocardiogram.

*Procedures:* - Colonoscopy is recommended to rule out colon cancer.

*Follow-up:*

- Await test results. Once received, a phone consultation will be scheduled to discuss the findings.

- If symptoms worsen, patient is advised to seek medical attention immediately.

Note: Always refer to the original conversation for a comprehensive understanding and in case of a need for any clarifications.

**Version 3:**

**SOAP Note**

**S:**

- Ash Park presents with a primary complaint of shortness of breath for approximately one month.

- Associated symptoms: Fatigue lasting several months, constipation, decreased appetite, unintentional weight loss (~10 lbs), and dark stools.

- Past medical history: Anemia (currently on iron supplements), high blood pressure (on lisinopril and amlodipine), high cholesterol (on rosuvastatin), type two diabetes (on metformin and pioglitazone), hypothyroidism (on thyroxine), leg cramps (taking magnesium supplements).

- Family history: Father died of a heart attack in his 50s, mother had type two diabetes and recently passed away in a car accident, older brother died from colon cancer.

**O:**

- Vitals: Temp 96.4°F, HR 170 bpm, BP 118/82 mmHg, RR 25/min, O2 sat 96% on room air, weight 220 lbs, height 5'1".

- Physical Examination:

* Patient alert, oriented, and in no acute distress.

* Respiratory: Clear lung sounds, no wheezing.

* Cardiovascular: Regular rate and rhythm, no murmurs.

* Gastrointestinal: Soft, non-tender abdomen, no masses.

* Musculoskeletal: No edema in extremities.

* Neurological: Ambulates with short steps and slightly curved posture.

**A:**

- Shortness of breath of unknown etiology.

- History of anemia with dark stools and a family history of colon cancer.

- Multiple chronic conditions (hypertension, hyperlipidemia, diabetes, hypothyroidism).

**P:**

1. Laboratory Tests:

* CBC to assess for anemia and other hematological disorders.

* Serum electrolytes, calcium, and magnesium.

* Kidney and liver function tests.

* TSH to evaluate thyroid function.

* Lipid panel.

* BNP to assess for potential heart failure.

2. Imaging and Diagnostics:

* Chest X-ray.

* EKG.

* Echocardiogram.

3. Referral to GI for colonoscopy to evaluate for potential colon cancer or other GI pathology.

4. Follow-up appointment to discuss test results and potential treatment options.

5. Advice given to seek emergency medical attention if experiencing worsening symptoms.

Note: Ash Park was provided with contact information for emergencies and follow-up questions.
